# Connected Through Mediated Social Touch: “*Better Than a Like on Facebook*.” A Longitudinal Explorative Field Study Among Geographically Separated Romantic Couples

**DOI:** 10.3389/fpsyg.2022.817787

**Published:** 2022-02-17

**Authors:** Martijn T. van Hattum, Gijs Huisman, Alexander Toet, Jan B. F. van Erp

**Affiliations:** ^1^Netherlands Organisation for Applied Scientific Research (TNO) Human Factors, Soesterberg, Netherlands; ^2^Faculty of Industrial Design Engineering, Delft University of Technology, Delft, Netherlands; ^3^Human Media Interaction, University of Twente, Enschede, Netherlands

**Keywords:** social touch, mediated touch, haptics, social connectedness, longing for touch, haptic bracelets, wearable haptics

## Abstract

In recent years, there has been a significant increase in research on mediated communication *via* social touch. Previous studies indicated that mediated social touch (MST) can induce similar positive outcomes to interpersonal touch. However, studies investigating the user experience of MST technology predominantly involve brief experiments that are performed in well-controlled laboratory conditions. Hence, it is still unknown how MST affects the relationship and communication between physically separated partners in a romantic relationship, in a naturalistic setting and over a longer period of time. In a longitudinal explorative field study, the effects of MST on social connectedness and longing for touch among geographically separated romantic couples were investigated in a naturalistic setting. For 2 weeks, 17 couples used haptic bracelets, that were connected *via* the internet, to exchange mediated squeeze-like touch signals. Before and after this period, they reported their feelings of social connectedness and longing for touch through questionnaires. The results show that the use of haptic bracelets (1) enhanced social connectedness among geographically separated couples but (2) did not affect their longing for touch. Interviews conducted at the end of the study were analyzed following the thematic analysis method to generate prominent themes and patterns in using MST technology among participant couples. Two main themes were generated that captured (a) the way the bracelets fostered a positive one-to-one connection between partners and (b) the way in which participants worked around their frustrations with the bracelets. Detailed findings and limitations of this longitudinal field study are further discussed, and suggestions are made for future research.

## Introduction

### Background

Our sense of touch plays an important role in interpersonal and affective communication (Knapp et al., 2013; Eid and Al Osman, 2016), as well as in human development, attachment, and wellbeing (Cascio et al., 2019). The sense of touch is also our primary way to communicate intimate emotions (Field, 2010; App et al., 2011). Social touch can serve to promote human wellbeing by relieving stress (Eckstein et al., 2020), an effect that can be effective at any age (Field, 2019), particularly for people in a romantic relationship (Huisman, 2017). Social touch can also enhance bonding between (romantic) couples (Gulledge et al., 2007) and improve the intimacy and quality of romantic relationships (Debrot et al., 2014).

However, social touch and its benefits are not always readily available. People can be geographically separated from one another for various reasons. Studies on the effects of social distancing on mental health during the COVID-19 related constraints found that deprivation of social touch was associated with higher levels of loneliness and anxiety and poorer overall psychological wellbeing and depression (Heidinger and Richter, 2020; Palgi et al., 2020; von Mohr et al., 2021). According to Beßler et al. (2019), a lack of touch results in longing for touch when the desire for touch outweighs the amount of experienced touch. A persistent lack of interpersonal touch (i.e., touch deprivation) can even cause various negative effects such as anxiety disorders and increased stress levels (Floyd, 2014), and may negatively affect relationships (Alsamarei, 2021). To prevent or counteract the negative consequences of touch deprivation, interpersonal touch should therefore ideally be readily available, even when people are physically separated.

The observation that interpersonal touch is essential for human wellbeing and communication has stimulated the development of mediated social touch (MST) technology, with the aim to enable affective haptic social interaction over a distance (Haans and IJsselsteijn, 2006; van Erp and Toet, 2015; Huisman, 2017; Ipakchian Askari et al., 2020a). Most studies investigating the user experience of MST technology involve brief (ranging from hours to at most a few days) experiments that are performed in well-controlled laboratory conditions (e.g., Rantala et al., 2013; Nakanishi et al., 2014; Erk et al., 2015; Ipakchian Askari et al., 2020b; Sykownik and Masuch, 2020; Price et al., 2022; for a review see Huisman, 2017). Although these studies provide valuable insights into the immediate perception of mediated touch signals, they do not reveal any long-term effects of MST-use, or whether the perception or use of MST changes over time. Two studies that investigated the way that romantic couples use MST technology over a longer period of time in naturalistic settings suggest that mediated touch can be experienced as meaningful (Saadatian et al., 2014) and can enhance feelings of connectedness (Park et al., 2013).

The work reported here covers a 2-week, longitudinal explorative field study into the effects of MST *via* haptic bracelets on the relation and communication between geographically separated romantic couples. The primary goal of this study is to examine whether the use of MST technology in a naturalistic setting and over a longer period of time affects the feeling of connectedness between geographically separated couples. The secondary goal is to explore how couples use the bracelets in a naturalistic setting (i.e., whether they develop certain communication patterns or attribute certain meanings to the signals).

### Related Work

Research on MST has culminated in the development of a wide range of prototype systems, such as Huggy Pajama (Teh et al., 2012), InTouch (Brave and Dahley, 1997), POKE (Park et al., 2013), Vibrobod (Dobson et al., 2001), and TaSST (Huisman et al., 2013) (for an extensive survey see Huisman, 2017). Previous research using these prototype systems shows mixed results in terms of replicating findings from unmediated social touch research (Ipakchian Askari et al., 2020b). Hence, it is currently not clear to what degree mediated touch can replicate the effects of unmediated social touch (Toet et al., 2013; van Erp and Toet, 2015). MST is typically not recognized as interpersonal touch (Ipakchian Askari et al., 2020a; Jewitt et al., 2020). It is also highly context dependent (Huisman, 2017; Ipakchian Askari et al., 2020a). Since MST can cause feelings of discomfort between strangers (Smith and MacLean, 2007), a closer (e.g., romantic) relationship may be preferred for this kind of tactile stimulation (Rantala et al., 2013; Suvilehto et al., 2015). Although currently available MST devices do not provide the emotional and contextual complexity of unmediated social touch, previous studies on MST still show some promising results. For instance, Bailenson et al. (2007) found that MST can communicate emotions to a certain degree, while others found that MST can induce increased feelings of intimacy and sympathy (Takahashi et al., 2011) and connectedness toward another person (van Erp and Toet, 2015). Also, a brief MST can induce prosocial behavior to the same degree as a brief unmediated touch (Haans and IJsselsteijn, 2009; Haans et al., 2014).

### Current Study

In this study we investigated how using MST technology for 2 weeks in daily life affects social connectedness and longing for touch among geographically separated romantic couples. For 2 weeks, 17 couples used haptic bracelets that were connected *via* the internet to exchange mediated squeeze-like touch signals. Before and after this test period, they reported their feelings of connectedness and longing for touch through questionnaires.

Various researchers emphasize the importance of social connectedness in (mediated) interpersonal communication. Social connectedness is described as “*a short-term experience of belonging and relatedness, based on quantitative and qualitative social appraisals, and relationship salience*” (van Bel et al., 2009, p. 1). According to Janssen et al. (2014), social connectedness is one of the most important needs in interpersonal relationships. A feeling of connectedness increases both physical and psychological wellbeing (Cacioppo and Patrick, 2008), and reduces loneliness (Janssen et al., 2014). Although social connectedness strongly relates to concepts such as loneliness and belonging, it differs from these in the way it is experienced. Social connectedness focusses on short-term experiences, whereas loneliness and belongingness reflect longer-term affective states (Visser et al., 2011). It has also been observed that MST can induce feelings of connectedness toward other persons (Wang et al., 2012; van Erp and Toet, 2015). van Bel et al. (2009) identified two types of social connectedness. At the overall level, social connectedness relates to a persons' entire social network, while it relates to a particular person at the individual level. To measure the effect of MST technology on social connectedness between geographically separated romantic couples in the present study, we focus on social connectedness on the individual level. Based on prior observations that the use of MST can result in increased social connectedness (Visser et al., 2011), our first hypothesis is:

Hypothesis 1: Geographically separated romantic couples will experience an enhanced feeling of social connectedness after using MST technology, compared to their feeling of social connectedness before using this technology.

The effect of MST on longing for touch was also investigated. Longing for touch can result from touch deprivation, and is described by Beßler et al. (2019) as a gap which is perceived when the frequency with which persons are being touched is lower than their touch wish. When a mediated touch is not recognized as unmediated social touch, MST cannot fulfill the need for touch and potentially alleviate the negative effects of touch deprivation. Moreover, MST could even enhance the desire for social touch if it makes the lack of ‘real,' unmediated social touch more salient. Hence, our second hypothesis is:

Hypothesis 2: After using MST technology, people that experience MST as interpersonal touch will experience less longing for touch, while people that do not experience MST as interpersonal touch will experience more or the same amount of longing for touch, than before using this technology.

In addition, some personal characteristics were measured that are known to influence the way people experience and respond to MST, like touch aversion, extraversion, and affinity with technology. For example, people who are touch aversive may experience relatively more negative consequences (such as anxiety) from MST compared to people who are not aversive to touch (Wilhelm et al., 2001). Another study, that compared mediated touch feedback to visual feedback, showed that more introverted people preferred touch feedback while more extravert people preferred visual feedback (van Erp and Toet, 2015). Lastly, people with low affinity for technology may experience mediated touch *via* MST technology more negatively than people with high affinity for technology (van Erp and Toet, 2015). These factors were taken into account in the design of the questionnaires used in the longitudinal study.

## Methods

### Participants

In this explorative study, as many participants as possible were recruited in the time frame of the study. A total of *N* = 17 couples, each consisting of one male and one female (34 participants in total), took part in the study. The age of participants ranged from 21 to 43 years (*M* = 26.82, *SD* = 4.96). The duration of the romantic relationship of the couples in this study varied between 2 and 57 months (*M* = 21.26, *SD* = 15.98). The couples were recruited through various (social) media platforms (Facebook, Instagram, LinkedIn, Proefbunny.nl), as well as the TNO database of volunteers. Inclusion criteria were (1) being in a romantic relationship while (2) not living together with the partner, (3) between 18 and 65 years old, (4) preferably having iPhones, and (5) English proficiency. All participant couples enrolled in this study around the same time of the year, with the last couple starting participation 10 days after the first couple. During the study, there were no (major) differences in COVID-19 related restrictions or other external factors that could influence the convenience or frequency of couples physically interacting with each other. See Supplementary Material J for all the inclusion and exclusion criteria for participants. The experimental protocol was reviewed and approved by the TNO Internal Review Board (Approval Ref: 2021-040) and was in accordance with the Helsinki Declaration of 1975, as revised in 2013 (World Medical Association, 2013). Participation was voluntary. All participants received a financial compensation of at least €40 (+ €5 bonus when filling in at least 75% of all questionnaires in the study). All participants gave their (digital) consent and were debriefed at the end of the study about the goal of the study.

### Materials

#### Hey Bracelets

**Figure 1:**
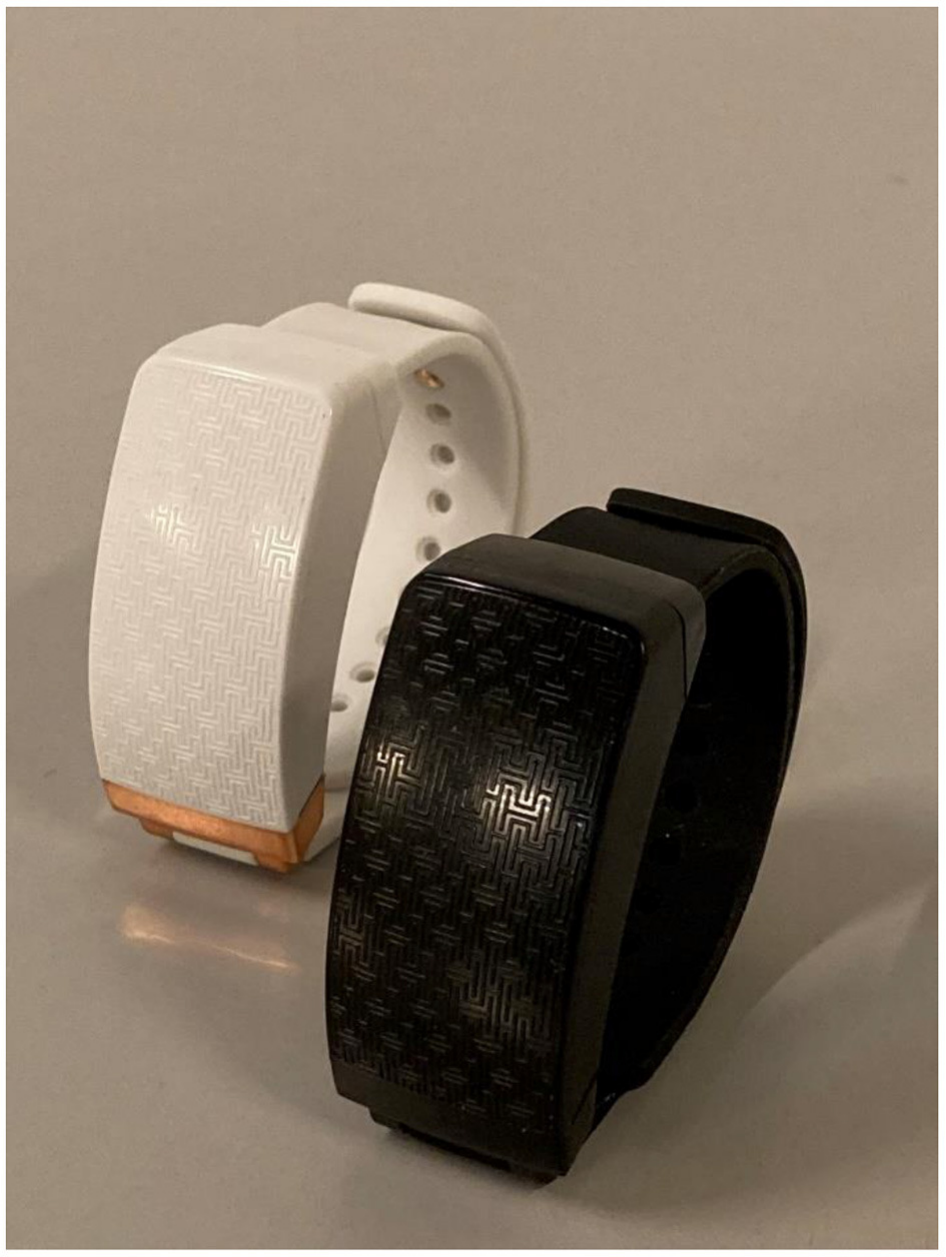
A pair of Hey bracelets.

The haptic devices used in this study are commercially available “Hey bracelets” (https://feelhey.com; see Figure 1). Hey bracelets are compatible with both Android and iOS devices and come with an app that allows bracelet pairs to be coupled *via* the internet. Users wear a Hey bracelet around their wrist. The bracelet uses internal sensors to detect when its surface is being touched. This touch is sent *via* Bluetooth from the bracelet to the Hey app running on the user's smartphone, which then transmits the touch *via* the internet to the Hey app running on the phone of a connected partner, which in turn activates the partner's bracelet. When activated, a Hey bracelet uses a 100 mA battery to power a small motor to pull part of the wristband into its casing. This contraction creates a squeezing sensation for the user. After the contraction, the motor loosens the wristband again until it has achieved its original position. Each time a ‘touch' signal is sent, the sender receives a vibration in the bracelet as a confirmation that the touch was sent.

Before starting the experiment, the authors extensively tested the Hey bracelets with both Samsung (Android) and iPhone (iOS) smartphones. It was found that the haptic bracelets functioned more reliably when paired with smartphones running iOS as an operating system compared to the Android operating system. It appeared that the battery management protocols on Android devices sometimes compromise the connectivity of the Hey bracelets. This finding resulted in the recruitment of participant couples that predominantly used iPhones. To minimize potential technical issues, the bracelets were manually updated to the latest firmware and fully charged before sending them to the participants.

#### Apps

Before using the haptic bracelets, participant couples were asked to install either two or three (depending on the operating system of their smartphones) apps for this study. These apps are described in the next sections.

##### Hey App

All participants installed the “Hey Bracelet” app on their smartphone (from Google's Play Store for Android phones and from Apple's App Store for iPhones). This app allows the bracelet to be connected to a mobile device *via* Bluetooth. The Hey app also enables two people to connect with each other *via* the internet through accounts with linked email addresses. In the “bracelet” tab of the Hey app, participants can see the status of their bracelet, such as the battery level and connection status. The connection status of a Hey bracelet is indicated by either a green dot (i.e., bracelet is connected to mobile phone *via* Bluetooth) or red dot (i.e., bracelet is not connected) in the upper right corner of the screen. *via* the “timeline” tab of the Hey app, participants can also (retrospectively) see when and from which location their partner sent them a ‘touch.' Participants received a personalized password for this app before the start of the study and were asked to login with their personal email address. All these details (e.g., email-addresses, passwords) were deleted at the end of the study.

##### HowAmI App

All participants installed the “HowAmI” app (an app developed in-house by TNO, available from Google's Play Store and from Apple's App Store). This app allowed participants to fill in questionnaires on their mobile device. The questionnaires used in this study were implemented in the programming language JavaScript Object Notation (JSON; https://www.json.org/json-en.html). The scripts for the questionnaires were uploaded to a secure (GDPR compliant) TNO server, which was connected to the HowAmI app. Each participant received personalized login details (username and password) for this app before the start of the study. All user data was deleted at the end of the study and no other (third) party had access to these data during the study.

##### DontKillMyApp App

Participants using Android devices were also asked to install the “DontKillMyApp” app, which provides information about battery management protocols. These protocols, which are typically quite persistent on Android devices, can compromise the usability of the Hey and HowAmI apps, as thereby the functionality of the haptic bracelets. The DontKillMyApp app is designed to tackle these battery management protocols on Android phones by showing users how to change their settings. This way, each device was correctly set-up to work with the bracelets and with the apps involved in the current study.

### Measures

#### Demographics

Prior to the experiment participants provided individual information (e.g., age, duration relationship, touch receptivity, personality, affinity for technology) through a first questionnaire that participants could fill in on the HowAmI app prior to receiving the bracelets (*Before Questions*).

#### Social Connectedness

To investigate whether the use of haptic bracelets enhances social connectedness among geographically separated romantic couples (Hypothesis 1), the individual version of the Social Connectedness Questionnaire (SCQ, van Bel et al., 2009) was applied. This scale contains 18 items that were rated on 7-point Likert scales: (1) *Completely disagree*, (2) *Disagree*, (3) *Somewhat disagree*, (4) *Neither agree nor disagree*, (5) *Somewhat agree*, (6) *Agree*, and (7) *Completely agree*. Social Connectedness is divided into five subscales/dimensions [relationship salience (RS), feelings of closeness (FC), shared understandings (SU), knowing each other's experiences (KE), and (dis)satisfaction with contact quality (CQ)], each with its own score. For this study, the “*X”* in the questions developed by van Bel et al. (2009) was replaced by “*my partner*.” All items of the dimension “*dissatisfaction with contact quality*” were reversed before analysis to make a high score contribute to (higher) social connectedness. The reliability (Cronbach's α) of the overall questionnaire was α = 0.93. The reliability (Cronbach's α) of the SCQ subscales were: RS, α = 0.84; FC, α = 0.86; SU, α = 0.82; KE, α = 0.87; CQ, α = 0.8.

#### Longing for Touch

To investigate whether longing for touch (dependent variable in this study) differs after the use of the bracelets (Hypothesis 2), the Longing for Interpersonal Touch Questionnaire (LITPQ) by Beßler et al. (2019) was used. Although this questionnaire was designed for various types of communication partners, only the specific romantic partner subscale of this questionnaire was used in the current study. Participants filled in the number of touches they experienced over the last 2 weeks, as well as the number of touches they wanted to experience with numbers ranging between 0 and infinity. The LITPQ score was then calculated by dividing the touch wish by the touch frequency, where LITPQ score > 1 = *longing for touch*, and LITPQ score <1 = *touch satisfied*.

#### Touch Avoidance

The Touch Avoidance Questionnaire (TAQ) by Ozolins and Sandberg (2009) was used to measure touch avoidance among participants. Only the questions specifically related to a (romantic) partner were used in this study (10 out of 37 questions total). Questions were answered on a 7-point Likert scale where (1) *Fully disagree*, (2) *Disagree*, (3) *Somewhat disagree*, (4) *Neither agree nor disagree*, (5) *Somewhat agree*, (6) *Agree*, and (7) *Fully agree*. Items 1, 5, and 6 were reversed before analysis to make a high score equivalent to a high level of touch avoidance. The reliability (Cronbach's α) of this questionnaire was α = 0.81.

#### Affinity for Technology

The Affinity for Technology Interaction (ATI) by Franke et al. (2019) was used to measure affinity for technology among participants. This questionnaire has recently been assessed through psychometric validation and was shown to be unidimensional, highly reliable, and to have high construct validity (Lezhnina and Kismihók, 2020). The ATI contains nine questions in total and was scored on a 6-point Likert scale: (1) *Completely disagree*, (2) *Largely disagree*, (3) *Slightly disagree*, (4) *Slightly agree*, (5) *Largely agree*, and (6) *Completely agree*. Items 3, 6, and 8 were reversed before analysis since these items were negatively worded. The reliability (Cronbach's α) of this questionnaire was α = 0.88.

#### Extraversion

To measure extraversion in this study, the Extraversion subscale of the International Personality Item Pool (IPIP) by Goldberg et al. (2006) was used. This questionnaire is based on earlier work by Goldberg (1992) and contains 10 questions in total. The questions were rated on a 5-point Likert scale: (1) *Very inaccurate*, (2) *Moderately inaccurate*, (3) *Neither accurate nor inaccurate*, (4) *Moderately accurate*, or (5) *Very accurate*. Items 2, 4, 6, 8, and 10 were reversed before analysis. The reliability (Cronbach's α) of this questionnaire was α = 0.88.

### Experimental Design

A within-subjects repeated measures design was used to evaluate the effects of the use of haptic bracelets on social connectedness and longing for touch among geographically separated romantic couples. *Social connectedness* and *longing for touch* were the two dependent variables in this study, while *time of measurement* (before/after using MST technology) was the independent variable. Connectedness and longing for touch were measured two times, once before using the bracelets (baseline measurement) and once after using the haptic bracelets. Individual user characteristics (e.g., affinity for technology, touch avoidance and extraversion) were measured as exploratory variables to explore their effect on the difference (post-score–pre-score) score of social connectedness.

### Procedure

The questions were presented in a fixed order in this study and were divided into three parts: the *Before Questions*, the *Daily Questions*, and the *After Questions* (see Table 1). The *Before* and *After Questions* served to measure the dependent variables (e.g., social connectedness and longing for touch) and the individual characteristics of the study sample, whereas the *Daily Questions* served to stimulate the involvement of participants in the study, and to reveal potential patterns in the use of the bracelets among couples. All questions were answered by participants in the HowAmI app on their own mobile device. Note, that the *Daily Questions* will not be considered further here because they were not directly relevant to the hypotheses, and response rates to these questions varied strongly between couples (see Section Limitations).

**Table 1:** Sequence of the three sections of questions as shown to participants in the HowAmI app.

**Before questions**	**Daily questions**	**After questions**
- Demographics - Duration Relationship - Social Connectedness (SCQ) - Longing for Touch (LITPQ) - Touch Avoidance (TAQ) - Affinity for Technology (ATI) - Extraversion (IPIP)	- Questions on use of the bracelet and physical interaction with partner	- Social Connectedness (SCQ) - Longing for Touch (LITPQ) - Explorative questions on the experience with and use of the haptic bracelets

#### Instructions

After signing up for the study, each participant received an email with instructions, an information document, and a digital informed consent form (see Supplementary Materials L, M). The instructional email informed participants that the experiment would take 2 weeks and that they could use the bracelets during this period in any way they liked. The primary goal of the study (the effects of haptic bracelets on social connectedness among couples) was not stated explicitly to avoid response bias. Instead, participants were informed that this was an explorative study into the use of haptic bracelets in a naturalistic field setting. Participants were asked to send back a signed version of the informed consent document and to confirm the address to which the bracelets should be mailed.

After returning their signed informed consent form, participants received an email with a confirmation that the bracelets were sent to them and with dedicated instructions for their particular mobile device, along with the bracelet manual, tips for using the bracelets, and login details for the Hey and HowAmI apps. Participants were also instructed to fill in the *Before Questions* before using the bracelets and each couple was given a “couple number” to pseudo-anonymize data before collection. Each couple was instructed to use the bracelets for 2 weeks, in any way (e.g., time and place) they liked, with the only requirement that they should actively use the bracelets during this period. Besides the instructions and information, participants were given the contact details of the experiment leader in case they encountered any problems, or if they had any questions before, during, or after the experiment.

After opening the HowAmI app for the first time, participants were shown an instructional text on the sequence of the questions and were again provided with the contact details of the experiment leader. When continuing, participants were presented with the *Before Questions* in the app and were asked to answer these questions.

#### Before Questions

The *Before Questions* were the first questions presented in the HowAmI app and contained questions that participants needed to fill in before using the haptic bracelets. The *Before Questions* were comprised of demographic questions and questions on social connectedness (van Bel et al., 2009), LITPQ (Beßler et al., 2019), TAQ (Ozolins and Sandberg, 2009), ATI (Franke et al., 2019), and IPIP (Goldberg et al., 2006). In this section, participants were also asked how long (in months) they had been in a relationship with their partner. The *Before Questions* served partly as a baseline measurement of social connectedness and longing for touch. This section contained a total of 63 questions and took around 9 min to fill in.

#### During the 2 Weeks of Testing

During the 14 days of the experiment, the participant leader contacted each couple at least once *via* telephone or email to ask if everything worked well (e.g., technical problems, filling in the questionnaires, etc.). This way, any potential technical issues could get tackled and at the same time participants were reminded to fill in the questionnaires. If participants had any questions or experienced any problems during these 2 weeks and reported those, the participant leader contacted them more than once. This applied to nearly half of all participant couples. This contact was done to keep the experienced burden for participants low, while keeping the involvement and response rate high.

#### After Questions

The *After Questions* were the last set of questions that participants needed to answer in the HowAmI app on their mobile device. These questions appeared 14 days after filling in the *Before Questions*, irrespective of the number of times participants answered the *Daily Questions*. The *After Questions* consisted of the Social Connectedness Questionnaire (van Bel et al., 2009), the Longing for Touch Picture Questionnaire (LITPQ: Beßler et al., 2019), and explorative questions on the experience with and use of the haptic bracelets (see Supplementary Material G). This section contained a total of 32 questions which took around 6 min to fill in. See Table 1 for the order of the three sets of questions in the HowAmI app.

#### End of the Experiment

After filling in the last questionnaire in the HowAmI app (the *After Questions*) the participants received an email to thank them for their participation. This email also included instructions to send back the bracelets through the return envelope that they had received at the beginning of the study, as well as an invitation for a semi-structured interview about their experience with the bracelets. Out of 36 participants, 32 (16 couples) agreed to take part in the interview. Each interview took approximately 30 min. The interview data were used to gain deeper insight into the way participants had used the bracelets (or potentially would have wanted to use them) and to explore potential patterns in the use of MST technology (secondary goal of the study).

### Data Processing and Analysis

#### Statistical Analysis

The quantitative data collected in this study was analyzed in IBM SPSS Statistics version 28.0 (www.ibm.com). Paired samples *t*-tests were conducted to investigate the effect of the use of MST technology for each couple on both *social connectedness* and *longing for touch*. Overall social connectedness per couple was calculated by averaging the scores of all 18 items per couple. Couples' scores on the subscales of social connectedness were calculated by averaging the aggregated scores of couples for each of the five subscales. Cohen's *d* (Cohen, 1988) was used to determine the magnitude of the effects found in this study. The individual scores for touch avoidance, affinity for technology, and extraversion were used to describe the current study sample, and to explore their relation to the social connectedness scores. Scatterplots and Pearson correlation coefficient (Pearson's *r*) were used to explore this relationship between participants' individual social connectedness scores and individual characteristics.

#### Thematic Analysis

For the analysis of the interview data, thematic analysis was used. The thematic analysis (TA) approach used here followed recommendations from Braun et al. (2018). The analysis aligns most closely with a ‘reflexive thematic analysis' approach (Braun et al., 2018; Braun and Clarke, 2019) where meaning is considered to be contextual and where researcher subjectivity is viewed as an asset in interpreting the data. This is in contrast to approaches that are more aligned with quantitative philosophies of qualitative data analysis (e.g., such as in content analysis or coding reliability TA; see also Braun et al., 2018). In the current analysis, the researchers followed an inductive approach to theme development where the analysis started from the data and where the final themes are the output of the analysis procedures. Where this TA approach deviated from the typical reflexive TA, is in the fact that multiple authors contributed to coding and theme development (though, see Braun et al., 2018, p. 852 and further) discussion of the role of multiple authors in reflexive TA), not to reach consensus, but rather, to build on each other's perspectives to gain greater insight into the data.

Initial coding was done by MvH and codes were further refined independently by AT and GH. Discussions between the researchers served to develop an initial set of themes, which were outlined in a number of thematic maps to further discuss the constructed themes and their connections. Between the construction of subsequent and refined thematic maps, the researchers reread the data to continuously check the salience and fit of the themes with the data, and to check whether the themes captured patterns of meaning across the dataset. This way, an initial set of four themes was reduced to two main themes.

## Results

### Social Connectedness

Social connectedness among 17 geographically separated couples was measured two times in the current study on a 7-point Likert scale: before and after using haptic bracelets. Potential answers on this scale ranged between 1 (i.e., low social connectedness) and 7 (i.e., high social connectedness). The obtained data was subsequently analyzed through a paired samples *t*-test (α = 0.05). This analysis was repeated for each dimension of social connectedness [i.e., relationship salience (dis)satisfaction with contact quality, shared understandings, knowing each other's experiences, and feelings of closeness] to investigate whether some dimensions of social connectedness were more affected by the use of MST technology than others.

#### Overall Social Connectedness

In the current study, 34 participants (17 couples) rated a total of 18 questions on Social Connectedness. A statistically significant difference was found between couples' social connectedness scores after (*M* = 5.67, *SD* = 0.64) using MST technology than before (*M* = 5.38, *SD* = 0.72) using MST technology, 95% CI [−0.45, −0.13], *t*_(16)_ = −3.77, *p* = 0.002. Cohen's *d* for this paired samples *t*-test was −0.42, which can be described as a small to medium effect size. This finding supports the first hypothesis of this study: *Social connectedness among geographically separated romantic couples will increase after using MST technology compared to social connectedness before using MST technology*.

#### Relationship Salience

Seventeen couples rated a total of 4 questions on relationship salience (RS) as dimension of social connectedness. The salience scores obtained after using MST technology (*M* = 5.77, *SD* = 0.62) were significantly higher than the scores obtained before using this technology (*M* = 5.29, *SD* = 0.77), 95% CI [−0.74, −0.21], *t*_(16)_ = −3.83, *p* = 0.001. Cohen's *d* for this paired samples *t*-test was −0.69, which can be described as a large effect size.

#### (Dis)Satisfaction With Contact Quality

Thirty-four participants (17 couples) rated a total of 3 questions on dissatisfaction of contact quality (CQ) as a dimension of social connectedness. Before analysis, the scores on all items of this dimension were reversed to make high scores contribute more to overall social connectedness. A statistically significant increase was found in couples' CQ scores after (*M* = 6.03, *SD* = 0.82) compared to before (*M* = 5.69, *SD* = 0.8) using MST technology, 95% CI [−0.57, −0.11]; *t*_(16)_ = −3.14, *p* = 0.006, with a small to medium effect size, *d* = −0.42.

#### Shared Understandings

Seventeen couples rated a total of 3 questions on shared understandings (SU) as a dimension of social connectedness. No statistically significant difference was found between couples' SU scores before (*M* = 5.32, *SD* = 0.76) and after (*M* = 5.51, *SD* = 0.79) using the haptic bracelets, 95% CI [−0.43, 0.062]; *t*_(16)_ = −1.59, *p* = 0.13.

#### Knowing Each Other's Experiences

Thirty-four participants (17 couples) rated a total of 4 questions on knowing each other's experiences (KE) as a dimension of social connectedness. No statistically significant difference was found between couples' KE scores before (*M* = 4.88, *SD* = 1.01) and after (*M* = 5.09, *SD* = 0.9) using the haptic bracelets, 95% CI [−0.52, 0.91]; *t*_(16)_ = −1.48, *p* = 0.16.

#### Feelings of Closeness

**Figure 2:**
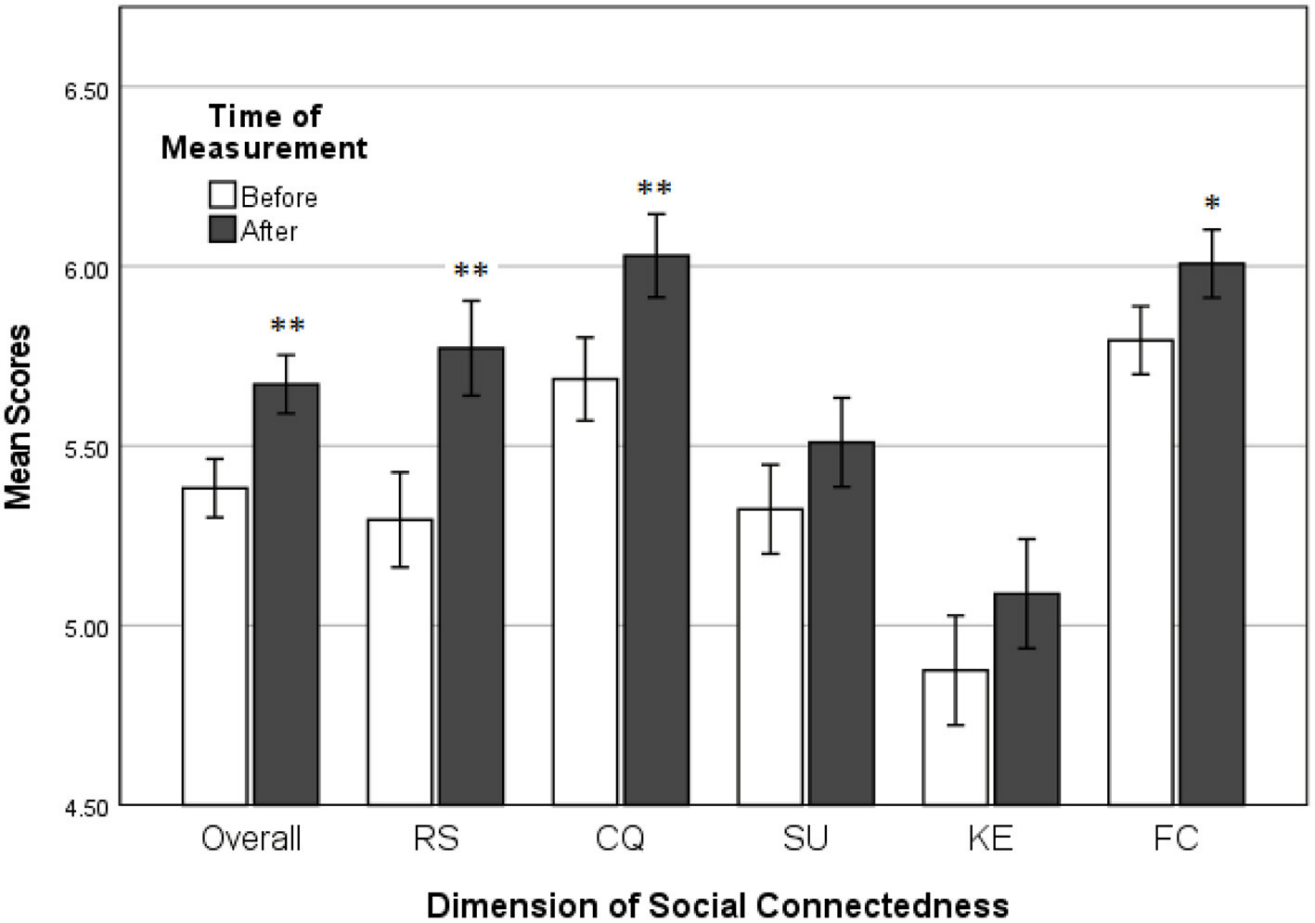
The mean scores of couples on the Social Connectedness Questionnaire, as a function of time of measurement (before and after using MST technology). Answers ranged from 1 (low social connectedness) to 7 (high social connectedness). α = 0.05. Couples' scores on all dimensions of the SCQ increased after 2 weeks of using MST technology. Significant increases are indicated by asterisks, where *p < 0.05; **p < 0.01.

Seventeen couples rated a total of 4 questions on feelings of closeness (FC) as a dimension of social connectedness. The couples' salience scores were significantly higher after using the MST technology (*M* = 6.01, *SD* = 0.73) than before (*M* = 5.79, *SD* = 0.84), 95% CI [−0.4, −0.23], *t*_(16)_ = −2.38, *p* = 0.03. Cohen's *d* for this paired samples *t*-test was −0.27, which can be described as a small effect size. Figure 2 shows the mean scores of participant couples on overall social connectedness, relationship salience, contact quality, and feelings of closeness, before and after using MST technology.

### Longing for Touch

A paired samples *t*-test (α = 0.05) was used to compare the 17 couples' longing for touch (LITPQ) scores before and after using the haptic bracelets. All 34 participants were asked to report the number of touches (ranging from 0 to infinity) they received from their partner over a period of 2 weeks (touch frequency), as well as the number of touches they wanted to receive from their partners (touch wish). A LITPQ score was then obtained by dividing the touch wish by the touch frequency. Raw data showed that before using MST technology, 73.53% of all participants in the current study sample had a LITPQ score > 1, which indicates longing for touch. On the other hand, 8.82% of the participants had a LITPQ score <1 (i.e., touch satisfied) and 17.65% had a LITPQ score of exactly 1 (i.e., touch wish and touch frequency were equal). After using MST technology for 2 weeks, 76.47% of all participants had a LITPQ score > 1, whereas 14.71% of participants had LITPQ scores <1 and 8.82% had a LITPQ score of 1. Noteworthy was that none of the participants experienced the signal of the Hey bracelet as interpersonal touch.

The Shapiro-Wilk statistic and normal Q-Q plots were used to test the assumption of normality. The assumption for a paired samples *t*-test was violated both these statistics. Moreover, descriptive statistics showed extreme LITPQ score values for four couples (couples 4, 7, 12, 13, all high outliers). First, the data for the LITPQ scores were logarithmically transformed (Log10) to control for these outliers. After the data was transformed, the LITPQ scores were still not normally distributed based on the Shapiro-Wilk statistic (*p* < 0.05) and normal Q-Q plots. After excluding the four couples with extreme LITPQ score values, the LITPQ data was normally distributed based on the Shapiro-Wilk statistic and normal Q-Q plots and there were no further outliers. For this trimmed data set (*N* = 13), there was no significant difference between the mean before (*M* = 1.32, *SD* = 0.28) and after LITPQ scores (*M* = 1.57, *SD* = 0.64), 95% CI [−0.55, 0.51]; *t*_(12)_ = −1.807, *p* = 0.096.

To assess the size and direction of the relationship between couples' social connectedness and longing for touch, Pearson's correlation analysis (Pearson's *r*) was executed. First, the difference scores (couples' post-score–pre-score) of both variables were calculated. Shapiro-Wilk tests suggested that scores were only normally distributed (*p* < 0.05) after excluding five (extreme) outliers (couples 4, 7, 9, 12, 13) from the original dataset of 17 couples. A Pearson's correlation analysis for this reduced dataset indicated that there was a weak, positive correlation between the difference scores of couples' social connectedness and longing for touch, *r*_(12)_ = 0.12, *p* = 0.71, n.s. The (non-significant) correlation is shown in Supplementary Material P.

### Individual Characteristics

**Figure 3:**
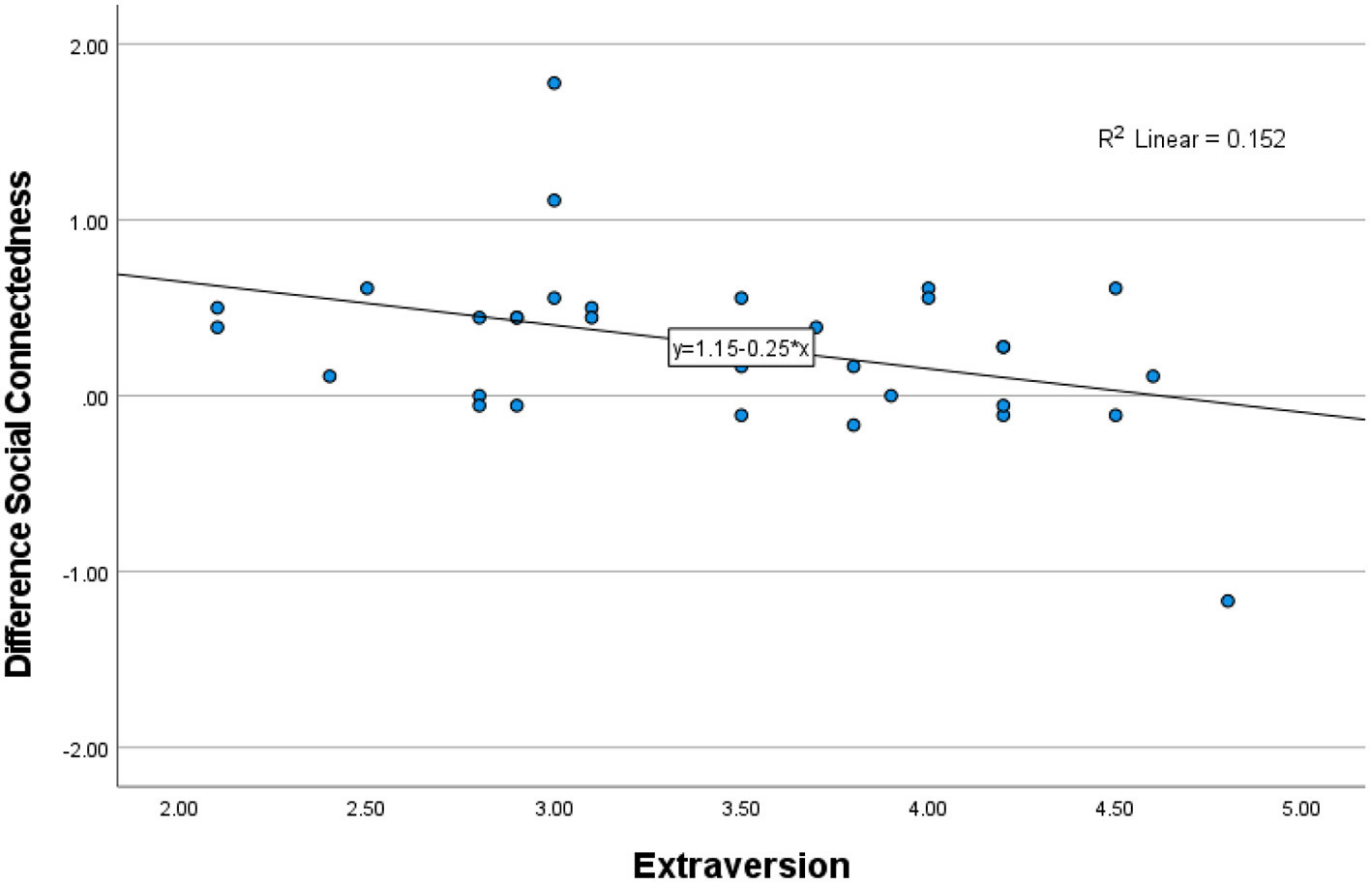
Participants' difference scores of overall Social Connectedness (post-score–pre-score), as a function of extraversion. The bivariate correlation was negative, with a medium effect size, *p* = 0.023.

To assess the size and direction of the linear relationship between the individual characteristics and the difference scores of social connectedness (post-score–pre-score), bivariate Pearson correlation coefficients (Pearson's *r*) were calculated. The bivariate correlation between the difference scores of social connectedness and *touch avoidance* was negative and weak, *r*_(32)_ = −0.19, *p* = 0.915, n.s. The bivariate correlation between the difference scores of social connectedness and *affinity for technology* was negative and weak, *r*_(32)_ = −0.127, *p* = 0.47, n.s. The bivariate correlation between the difference scores of social connectedness and *extraversion* was negative and medium, *r*_(32)_ = −0.39, *p* = 0.023. The significant (negative) correlation between social connectedness and extraversion is shown below in Figure 3.

### Thematic Analysis of Interviews

**Figure 4:**
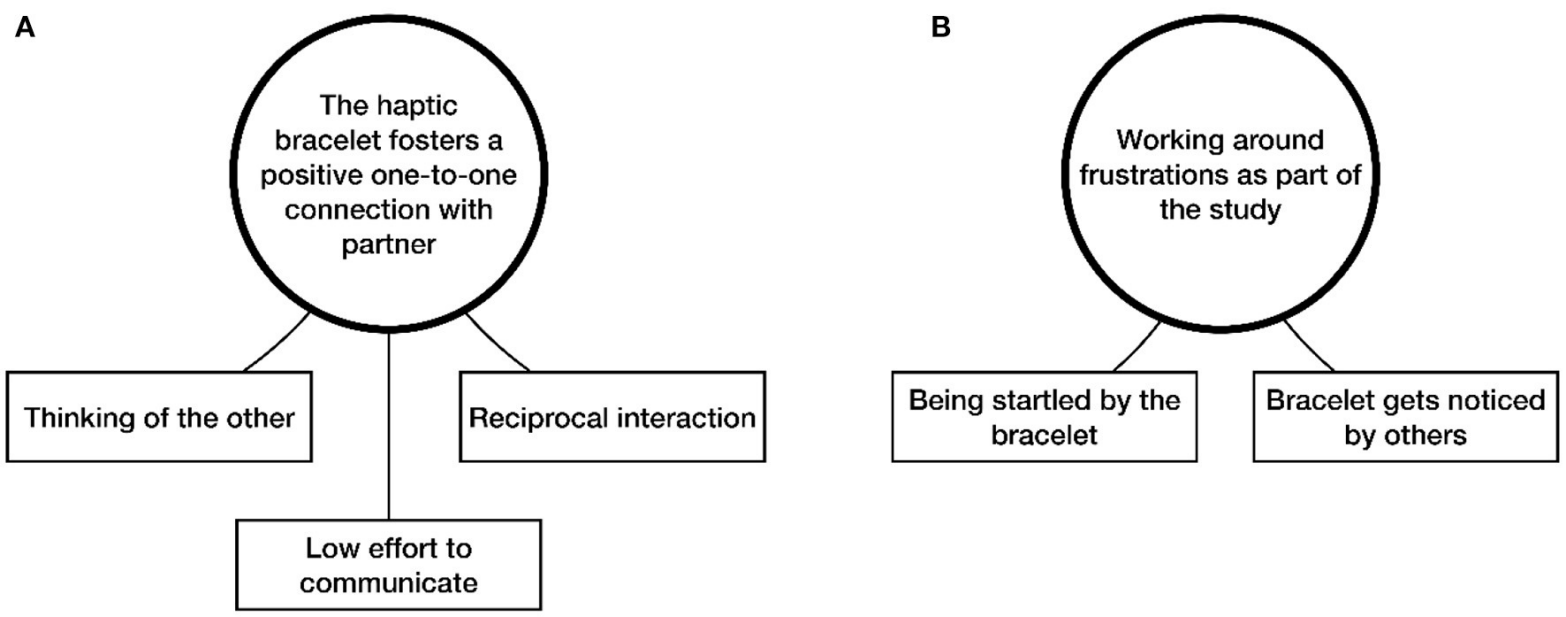
Final thematic map, showing the two main themes derived from thematic analysis. **(A)** The first main theme with its three subthemes and **(B)** the second main theme and its two subthemes.

The two main themes that were generated through the TA are depicted in the final thematic map (see Figure 4). The two main themes relate to participants' accounts of their use of the haptic bracelets during the study: (1) *The haptic bracelet fosters a positive one-to-one connection with the partner*; and (2) *Working around frustrations as part of the study*. The first main theme has three subthemes, the second main theme has two subthemes.

The quotes that are used from the data were translated from Dutch into English. All original quotes can be found in the Supplementary Material R. Names, as well as place names are anonymized (“NAME” and “CITY,” respectively) in the quotes. Quotes are labeled per couple number and participant number within the couple (either 1 or 2).

#### Theme 1: The Haptic Bracelet Fosters a Positive One-to-One Connection With Partner

The first main theme *The haptic bracelet fosters a positive one-to-one connection with partner* captures how participants talked about the use of the haptic bracelet as enabling them to establish a one-to-one connection with their partner. Communication through the bracelets was meaningful for participants because they knew the signal came from their partner, and not because it was a physical signal (i.e., haptic feedback) *per se*. Some participants explicitly described this one-to-one connection in their accounts of the use of the bracelets:

“*I was very aware of wearing the bracelet and what happens at the moment it activates, so I couldn't really compare it to real touch. However, the thought behind it is what makes it nice, that I know that she did it, that she sent it, so it is more the contact that you have. It is what is behind it that makes it nice for me*.” (Couple 18, PP2)

“*Yes, I have to say because you know that the touch comes from your partner, that is how you imagine it is a touch*.” (Couple 1, PP1)

“*Also, the fact that you know that no-one else does this. Your phone can vibrate because of someone else. Here, you just know for sure that it was NAME. That is a nice feeling*.” (Couple 3, PP2)

Here, participants explicitly referred to how they interpreted the received signal and the fact that it represents a direct connection with their partner that makes it meaningful for them. For other participants, this one-to-one connection with their partner was implied in their description of the use of the haptic bracelets. For some, being separated from their partner by physical distance brought this notion of connection more to the surface:

“*Yes, and definitely also because normally during the period that we used the bracelets he was a lot in CITY1 and I was a lot in CITY2, so you really miss each other and you miss being able to hug each other or give each other a kiss every now and again. And this was a way to still feel close. The first time I didn't wear it after the study I was totally like, ‘now what'?”* (Couple 11, PP1)

Participants who implicitly or explicitly referred to the one-to-one connection with their partner often saw this as representing a way of ‘thinking of the other.' This subtheme had very high prevalence in the data. Virtually all couples at one point or another mentioned that ‘thinking of' was what the interaction through the bracelets meant to them:

“*For me, it didn't just mean 'I am here' but especially 'I am thinking of you'. She knows I am thinking of her, that I am preoccupied with her rather than myself. I think that that is very nice. If someone takes the time in between everything else, no matter how busy your day is, to send 'I am thinking of you'. Nothing more. I think that that is very, very nice*.” (Couple 18, PP2)

Interestingly, there were no clear mentions of using the bracelets for more complex or elaborate ways of encoding messages (e.g., two squeezes means ‘I love you'). With this it would seem the bracelets served as a way to enhance feelings of social connectedness (van Bel et al., 2009), rather than serve as a tactile communication device *per se* (Haans and IJsselsteijn, 2006):

“*In the beginning I was thinking; NAME has sent a touch so I send it back, but of course it is not some kind of Morse code, or visible communication. At one moment you quit doing that and it is more during moments that you think of the other that you send a touch*.” (Couple 17, PP2)

“*I don't actually connect it to touch that much. For me it was more, for example, when I woke up and NAME sent me a touch I thought 'my partner is thinking of me, that's nice' and not a touch per se*.” (Couple 10, PP1)

In some instances, it was not just the notion that when a touch was received that participants felt that this represented their partner thinking of them. In some cases, they would actively demand or request that their partner think of them by sending a touch signal themselves:

“*I especially appreciated it when I was feeling down that you can ask for attention by sending [a haptic signal], like 'I just need some attention from you right now'*.” (Couple 11, PP1)

“*I actually felt like receiving a touch, but I didn't get one. So then I would send one myself and get one back. Kind of like asking for attention*” (Couple 6, PP2)

This idea of ‘demanding attention' connects with the fact that in most cases the sending of a haptic signal also resulted in receiving a signal in return, and vice versa. In other words, often, the interaction was reciprocal, involving a back-and-forth of sending and receiving the haptic signals. This was not just the case when participants would ‘demand attention,' but also more generally, participants expressed that they felt that it would be strange not to send a haptic signal back when receiving such a signal themselves:

“*Nine out of ten times I would send one back*.” (Couple 3, PP1)

“*It felt very weird not to send one back*.” (Couple 9, PP1)

Participants actively considered the reciprocal nature of the interaction in the one-to-one connection that the bracelets enabled, and this impacted the way they would use their bracelet. One participant captured the way that the subthemes of ‘thinking of' and reciprocity of the interaction relate to each other as follows:

“*Sometimes I wanted to let him know that I was thinking of him and at other times I wanted attention myself. I would wish he would send me something, so then I would send him a touch and then I hoped he would send something back. I noticed that I found it difficult if that did not happen, because I really wanted to receive something back. But perhaps someone is busy at that moment*.” (Couple 16, PP1)

The fact that it did not take a lot of effort to communicate with one's partner, was a final element that had high prevalence in the data and that was important to the fostering of a positive one-to-one connection through the haptic bracelets. Many participants described how the haptic bracelets allowed for a simpler or more direct interaction compared to, say, smartphone messaging services or video chat:

“*The moment that you are thinking of someone, you don't have to look at your phone or anything. So that kind of ease-of-use is there*.” (Couple 12, PP2)

“*I thought it was quite useful when you don't have the time to write a message, because you have to think more about writing a message. You can just put your hand on the bracelet to let someone know you are thinking of them. I thought that was a really nice added value*.” (Couple 16, PP1)

One participant likened this low-effort way of communicating to functionalities on a popular social media platform:

“*Better than a like on Facebook*.” (Couple 16, PP2)

The low-effort, simple, and more direct interaction through the haptic bracelets was especially appreciated by participants because it would allow for the one-to-one connection to be maintained even in situations where other forms of mediated communication would be more difficult to use. Examples provided by participants of such situations include being busy at work, or being in the company of other people:

“*When I don't have time to send a text message, or if I'm busy at work. I work with people, with guests, and so I cannot really pick up my phone then. In those situations, sending a touch is just a little easier than sending a text message*.” (Couple 5, PP1)

“*No, I don't just pick up my phone to send a text message. It is much easier to just put your hand [on the bracelet]. You could also just do that while talking with someone. You could sneakily put your hand on your wrist*.” (Couple 3, PP2)

Descriptions of the use of the bracelet in combination with other media, such as text messaging, was also predominant in the data. However, use of other media was most often discussed in such a way that the bracelet served as a low effort way to maintain the one-to-one connection with the partner. Text messaging was then sometimes used to write a more elaborate message, or to check-in with the partner when a touch was not reciprocated.

To summarize, the first main theme captures how participants, implicitly or explicitly, talked about their use of the bracelet as fostering a positive one-to-one connection with their partner. The haptic bracelets were not so much used for haptic messaging, but mainly served as a low-key way to let the other know ‘*I'm thinking of you'*. Most participants when sending a haptic signal expected to receive a signal back from their partner. Conversely, when receiving a signal, participants described how they felt obligated to respond. The fact that the bracelet could be used without reaching for one's phone was experienced as positive by participants and influenced the way the bracelets were used in situations such as during work or when being with other people.

#### Theme 2: Working Around Frustrations as Part of the Study

The second main theme *Working around frustrations as part of the study* captures how the use of the haptic bracelets also led to annoyances or frustrations for participants. In the data there was a high prevalence of remarks pertaining to such frustrations with the use of the bracelets. However, while participants expressed their frustrations, in almost all cases they also detailed how they found ways to work around these frustrations. The researchers see this as stemming from the fact that participants were well aware that they were taking part in a study and did not want to drop out or disappoint the researchers. Some participants mentioned this literally:

“*At one moment it almost become a burden to start using [the bracelet] because we were having issues with the Bluetooth connection. But, of course, we also kind of did it for you. We knew we were part of a study so that lead to the use [of the bracelets] becoming a bit messy*.” (Couple 16, PP1)

Not all participants explicitly described being part of the study as the underlying reason for finding solutions to the problems they encountered, despite experiencing frustration. Nearly all participants exhibited an attitude of ‘let's make this work.' This attitude was apparent in many descriptions and for many issues that participants encountered, including Bluetooth connectivity issues, issues with the bracelet falling off of participants' wrists, accidentally sent touches, and excessive noise production by the bracelets. While the experienced issues as sources of frustration may have been diverse, the attitude of working around the frustrations was shared by most participants. This attitude also encompassed ways in which participants made sense of the interaction despite issues arising, such as in relation to receiving accidental touches:

“*I didn't mind [accidental touches] so much. At a certain moment I started taking it into account. Sometimes you receive one [intended] touch and at other times you receive many accidental touches, but do you know what? For me that one [intended] touch still outweighed the other nine that were perhaps accidental because there was contact in that one moment, it's not like the bracelet is on the table sending touches on its own.”* (Couple 18, PP2).

One specific frustration with high prevalence in the data that participants described finding ways to deal with, relates to the fact that the activation of the bracelet startled them, in one particular case to the extent that it led to spilling coffee:

“*Every time [the bracelet] went off it startled me badly. On the first day I spilled a cup of coffee over my keyboard because I was so startled*.” (Couple 18, PP1)

Similar remarks of being startled by the bracelet activating were made by other participants. In most cases participants ascribed this to a combination of the sound produced by the bracelet as well as the unexpected nature and unfamiliar feeling of the activation. Here, the novelty of the device and the lack of experience with similar types of haptic communication devices prior to this study, most likely contributed to the startling reactions to the bracelet activating. In all cases, however, participants adopted an attitude of ‘dealing with it,' which in this case meant a conscious process of familiarization and acclimatization (i.e., being conscious of the time it takes to get used to the bracelet activating):

“*There were a few times where [the bracelet] really startled me. It has a kind of silent mode, but it took a few days for me to discover that. I did wear it to work and even during some meetings it really startled me when that thing went off. After a while you get used to it, and you think 'oh, it's that app', but the sound volume and the unexpectedness of it are a bit strange still*.” (Couple 17, PP2)

This conscious effort also meant that when being startled by the bracelet, even when only a little, participants were taken out of their concentration or briefly distracted from other things that they were doing. This was described as a related, minor annoyance:

“*When I'm fully absorbed in something I really want to focus and if that thing [the bracelet] then activates, I'm startled by it. You're all like 'hold on'. It is not for very long, but it does take some time to get back to what you were doing before*.” (Couple 3, PP1)

Related to this discussion of being startled by the bracelet was the fact that participants commented on other people present in the same space noticing the bracelet activating. In a few cases this was in relation to the wearer of the bracelet also being startled, but most often frustration with the bracelet and ways of working around this frustration, related to the notion of others noticing the bracelet. Examples given by participants of how others noticed the bracelet describe the size of the device, and, with high prevalence, the sound produced by the bracelet's activation. Depending on the situation, this led to more or less frustration experienced by participants. A clear way of working around this issue that many participants discussed was strategically deciding when not to wear the bracelet in situations where it could be noticed by others. For example, participants described it as being unprofessional when the bracelet activation would be noticed by others during a meeting, even during online meetings:

“*I wouldn't wear it during video calls, which is something you now do often for your studies. I wouldn't wear it because it would make a sound. If you're in a professional setting with your teacher and you hear that sound; no, I didn't use it then*.” (Couple 1, PP1)

“*I would take it off when I was in a meeting. That was more because I was thinking 'if it would go off now that would be awkward'*.” (Couple 4, PP2)

“*If I'm honest, it was really awkward with some people, some colleagues*.” (Couple 3, PP1)

The sound produced by the bracelet upon activation was a major factor in others noticing the bracelet and in subsequent feelings of unease experienced by participants. One participant expressed this by explaining how they were aware of others staring:

“*The sound the bracelets produce is not very discrete. When you're in a room with other people, everybody there also knows when you receive a touch. Everyone would be staring*.” (Couple 2, PP1).

From these accounts by participants, it can be seen that the bracelet did not just play a role as a mediating device between both partners, but that it was also a part of other social interactions, although, with more negative connotations. The fact that participants actively worked around the bracelet getting noticed by others shows that it was not properly embedded in existing social structures but that it, instead, had a disruptive effect. Again, the researchers would argue that the willingness of participants to work around this disruption is largely due to the fact that they were aware of being part of a study. The researchers also remark that haptic devices for social communication, such as the haptic bracelets used in this study, should not only be considered from the perspective of remote communication, but should be viewed within a larger context of social interactions that occur during the use of such devices.

To summarize, the second main theme captures how participants described diverse sources of frustration with the bracelets (including frustrations originating from technical issues) but that they, in nearly all cases, exhibited a willingness to work around their frustrations because they were aware of being part of a study. The initial novelty of the device combined with the sound production and unfamiliar squeezing sensation, meant that several participants were startled by the bracelet activating. Here, their remarks show a process of “*getting used to*” the bracelets. In addition, participants shared their frustrations with the bracelet when it was noticed by others and participants would work around this by strategically deciding when and where not to wear the bracelet.

## Discussion and Conclusion

The hypotheses of this longitudinal explorative field study were (1) Geographically separated romantic couples will experience an enhanced feeling of social connectedness after using MST technology, compared to their feeling of social connectedness before using this technology, and (2) After using MST technology, people that experience MST as interpersonal touch will experience less longing for touch, while people that do not experience MST as interpersonal touch will experience more or the same amount of longing for touch, than before using this technology. The results show that the use of haptic bracelets (1) enhanced social connectedness among geographically separated couples but (2) did not affect their longing for touch. Interviews conducted at the end of the study were analyzed by way of (reflexive) thematic analysis to generate two main themes (each with their own subthemes), reflecting the way participants talked about their use of MST technology during the study. These themes were (a) The haptic bracelet fostered a positive one-to-one connection with a romantic partner; and (b) Participants were willing to work around frustrations as part of the study. In the rest of this section, the findings and limitations of this study will be discussed in further detail, and suggestions will be made for future research.

### Social Connectedness

In agreement with our first hypothesis, the geographically separated romantic couples that participated in this study reported a significant increase of overall social connectedness levels after using the haptic bracelets in daily life for a period of 2 weeks. This result is also in line with similar findings from studies that were performed for a briefer period and in restricted (laboratory) conditions (Visser et al., 2011; Park et al., 2013; van Erp and Toet, 2015; Price et al., 2022).

The effects of the haptic bracelets were investigated on all five dimensions or subscales of social connectedness. This analysis showed that *relationship salience* increased significantly (with a large effect size) after using the haptic bracelets. This finding agrees with the results of Visser et al. (2011), who noticed increased levels of relationship salience after using a social awareness system called SnowGlobe. Relationship salience entails how prominent a relationship is in a persons' mind (Visser et al., 2011). In the current study, communicating ‘touches' *via* the haptic bracelets in addition to other ways of communication [e.g., (video-) calling, texting], may have reminded participants more frequently of their mutual relationship (thereby increasing its salience). This explanation aligns well with the first main theme of the TA, which describes the bracelets as fostering a one-to-one connection between the partners. Participants often remarked how they used the bracelets as a way to signal to their partners that they were thinking of them. Conversely, they also often stated that they interpreted the reception of a haptic signal as a sign that their partner was thinking of them.

Furthermore, a significant increase in *feelings of closeness* (as a dimension of social connectedness) was found among couples after using MST technology. Feelings of closeness entails the social presence of another person in one's mind (Visser et al., 2011). Like the increase in salience, this significant increase in closeness is also consistent with prior research (Visser et al., 2011), and may stem from the way the haptic bracelets were used in this study: the use of MST technology in addition to the existing communication channels of couples [such as (video-)calling] may have increased the perceived social presence of a partner (thereby increasing feelings of closeness). Lab-based research by Price et al. (2022) indeed indicates that MST technology can contribute to a feeling of ‘tactile presence' where the technology signals that the other ‘is there.' Again, this aligns closely with results of the TA, in particular the first main theme.

Although a significant increase in both dimensions after using MST technology was observed, the increase in closeness had a small effect size, whereas the increase in salience had a large effect size. This difference may be related to the baseline levels of the average scores on these two subscales of social connectedness: the average scores on *feelings of closeness* were higher (5.79 on a 7-point Likert scale) than the *relationship salience* scores (5.29) before using MST technology. MST probably does not contribute strongly to *feelings of closeness* between the dyads that already experience high levels of closeness. Future studies should investigate the effect of (similar) MST technology on these dimensions of social connectedness among dyads with other relationships (not romantic; friends or acquaintances).

*Satisfaction with contact quality* (CQ), as subscale of social connectedness, also showed a significant increase (with a small to medium effect size) after using MST technology. Asking (*After Questions*) the participants how the haptic bracelets fit in their other ways of communication (see Supplementary Material G), 75% of all participants rated them as complementary. On the other hand, 21.9% of all participant found the bracelets not adding anything to existing communication, and 3.1% thought the bracelets could replace their existing ways of communication. This illustrates that the majority of participants in this study think this form of MST technology compliments their other ways of communication, instead of seeing this technology as a replacement or that it has no added value. One subtheme in the first main theme from the TA outlines how the bracelets were mainly described as a low-effort way to communicate in comparison to other technologies. Participants described how this enabled them to stay connected in situations where, for example, using their smartphone was more difficult (e.g., while being busy at work). In these situations, as mentioned by participants, the bracelets complemented their use of other technologies and media.

Analyses of the other two subscales of social connectedness, *knowing each other's experience* (KE) and *shared understandings* (SU), showed no significant difference between couples' scores before and after using MST technology. These findings may be explained by the specific MST technology (haptic bracelets) used in this study. This MST technology was tested in isolation, without any other (mediated) sensory input. Moreover, the haptic bracelets only conveyed a single bit of communicative information, which was a mediated touch signal giving a squeezing sensation. As Kaye (2006) argued, a low bandwidth signal (such as produced by the haptic bracelets) leaves a lot of room for interpretation within pre-existing relationships. At the same time, the bracelets' signal does not convey the experiences or understandings of another person. As such, KE and SU may not be affected by MST technology when implemented in an isolated fashion, and perhaps a more multimodal approach of testing this kind of technology (e.g., combined with mediated audio/visual cues) may influence these dimensions of social connectedness. Work by Price et al. (2022) underscores this notion and illustrates how multimodal *haptic* signals (e.g., temperature) could also play a role here. Still, the *thinking of the other* subtheme from the TA illustrates how, even with a low-bandwidth signal, and lack of other (haptic) modalities, participants ascribed specific meaning to receiving a haptic signal through the bracelets and had specific intentions when sending signals.

What can be concluded from this study is that overall social connectedness among geographically separated romantic couples increased after using MST technology, and that the dimensions contributing most to this increase were relationship salience, feelings of closeness, and contact quality. As such, the first hypothesis of this study (*Geographically separated romantic couples will experience an enhanced feeling of social connectedness after using MST technology, compared to their feeling of social connectedness before using this technology*) was supported.

### Longing for Touch

Analysis of the scores on the Longing for Touch Picture Questionnaire (LITPQ, Beßler et al., 2019) showed no difference in longing for touch among geographically separated couples before and after using MST technology for 2 weeks. More specifically, after deleting outliers (4 couples) from the total dataset to correct for the violation of normality, no difference was found in the average LITPQ scores among 13 couples before and after using the haptic bracelets. This finding is consistent with the distribution of participants that experienced longing for touch (by having a LITPQ score > 1) across the different times of measurement in this study. Nearly 80% of all participants indicated longing for touch before using the haptic bracelets, as their touch wish outweighed their experienced touch. Although LITPQ scores changed for some participants after using MST technology, the percentage of participants with LITPQ scores > 1 remained nearly identical after using the bracelets (73.5% before vs. 76.5% after), indicating no significant change in longing for touch among participant couples.

What should be noted is that there was high variability across the LITPQ scores of couples, and the distribution of scores was not normally distributed among the 17 participant couples. After deleting the most extreme outliers, the LITPQ was normally distributed. A potential explanation for the high variability in LITPQ data is the way in which the LITPQ is scored by participants: the possible answers that could be given on the amount of wished and experienced touches were between 0 and infinity. Furthermore, the LITPQ is fairly recently developed (in 2020) and thus the instrument has not been elaborately validated yet. However, as prior research indicated, there is only a limited number of validated instruments that aim to measure a lack of touch (Punyanunt-Carter and Wrench, 2009). The Longing For Touch Picture Questionnaire (LITPQ, Beßler et al., 2019) was specifically chosen for this study due to the addition of the specific partner subscale within the questionnaire. Other instruments, such as the Touch Deprivation Scale (Punyanunt-Carter and Wrench, 2009), do not include specific questions or sections relating to measuring touch deprivation among (romantic) dyads. As such, the LITPQ seemed a more viable instrument to utilize in the current study.

Furthermore, questions at the end of the study (*After Questions*) revealed that none of the participants experienced the signal (squeeze) of the haptic bracelets as interpersonal touch. This finding agrees with other recent studies that found that mediated touch is typically not experienced as unmediated social touch (Ipakchian Askari et al., 2020a; Jewitt et al., 2020). Again, this aligns with the first main theme and subthemes of the TA. Participants described the use of the bracelets more as a low-effort way to signal to the other that they are thinking of them. No clear mentions of the bracelets actually being used as a mediated social *touch* device were present in the interview data.

Both the analysis of the LITPQ data and the fact that participants did not recognize the haptic bracelets' signal as interpersonal touch suggest that longing for touch among geographically separated couples is not affected by the use of haptic bracelets as MST technology. Thus, hypothesis 2 (*After using MST technology, people that experience MST as* interpersonal *touch will experience less longing for touch, while people that do not experience MST as* interpersonal *touch will experience more or the same amount of longing for touch, than before using this technology*) was not supported.

### Individual Characteristics

Analyses of the individual characteristics data showed that, on average, the current study sample scored relatively low on *touch avoidance* (1.93 on a 7-point Likert scale), high on *affinity for technology* (4.11 on a 6-point Likert scale), and above average on *extraversion* (3.44 on a 5-point Likert scale, indicating more extraverted than introverted characteristics within participants). These findings may be explained by the recruitment protocol of the study, as a specific sample was recruited (e.g., couples not living together and both preferably having iPhones). The recruitment flier (see Supplementary Material K) and information document (see Supplementary Material L) may have attracted couples that already scored low on touch avoidance (especially related to partner-specific touch) and high on affinity for technology (as the flier and other information indicated working with a new technology for 2 weeks). It seems likely that people with these traits are more inclined to (voluntary) participate in a study that involves touch and technology. The fact that participants scored relatively high on *affinity for technology* could explain the high prevalence of remarks in the interviews that in the TA were taken as an attitude of *working around frustrations* (the second main theme). Their interest in technology could mean that participants were more eager to figure out how to make the bracelets work properly during the study.

Explorative scatterplots and correlations of participants' individual characteristics and social connectedness scores revealed a significant negative correlation between overall social connectedness and extraversion. This may indicate that more introverted participants had a bigger increase in social connectedness after using MST technology, compared to more extraverted participants in the present study. This effect is similar to findings in prior research on MST (Erk et al., 2015). Correlations between participants' overall social connectedness scores and touch avoidance/affinity for technology did not yield significant results. A potential explanation for these results may again be found in the sample of the present study. The effects of MST technology were observed for dyads in a romantic relationship, while non-romantic dyads have not been tested. The interpretation of mediated touch may be less (negatively) affected by high touch avoidance or low levels of affinity for technology among romantic dyads, compared to non-romantic dyads (Rantala et al., 2013; Suvilehto et al., 2015).

### Limitations

A major limitation of this study were the technical problems that a majority of participants experienced, to a more or lesser extent, when using the haptic bracelets. Even though the haptic bracelets were elaborately tested before the start of the study, and several instructions and tips were drawn up specifically tailored to the phones of participants, these issues still persisted. Overall, nearly 15% of all participants needed to receive a new bracelet over the course of the study due to technical issues. The problems that participants encountered can largely be divided into two categories: either ‘touches' were sent unintentionally (e.g., touches were sent when sitting with arms crossed), or intentionally sent touches were not physically received by the partner. The latter issue was mainly caused by cessation of the Bluetooth connection between the Hey bracelet and mobile phones of participants. See Supplementary Material O for a summary of the feedback of participants on the bracelets in the current study.

These technical issues also led to the generation of a main theme in the TA capturing how participants adopted an attitude of working around their frustrations with the bracelets. As outlined in the TA, there were several technical issues that contributed to participants experiencing frustrations and finding ways to deal with those frustrations by changing their use of the bracelets, at least in part, because they were well-aware of being part of an ongoing study. From this, it is clear that the technical issues had a significant impact on the use of the bracelets (e.g., strategically deciding when not to wear the bracelets) within the scope of the study and, thus, the results reported here should be interpreted with this in mind. It could also be that the fact that the bracelets did not function flawlessly resulted in participants having more frequent contact through other means to resolve the issues with the bracelets. Though there was not a high prevalence of remarks to this effect in the interview data, it could still be that this increase in contact could potentially have affected participants' ratings on social connectedness.

Prior research has struggled with reporting quantitative data in longitudinal studies due to high percentages (>30%) of missing data (Visser et al., 2011). This was also the case in the present study. Although the before and after questions were completed by all 17 couples (34 participants), the current study struggled with receiving participants' answers on the daily questionnaires (nearly 40% missing data). While the daily questions were specifically designed to be easy to answer, participants apparently still struggled with answering them consistently. Part of this could be contributed, again, to technical issues experienced by the participants. Open-ended feedback in the explorative questions revealed that some participants did not want to answer the daily questions in the HowAmI app on days when the bracelets did not function properly.

Another limitation of the current study lies in the study sample. Initially, the aim was to recruit 20 couples. However, recruitment was challenging due to the strict recruitment criteria (see Supplementary Material J). Ultimately, as many participants were recruited as possible in the time frame of this study. In total, 18 couples participated in this study and analyses were conducted on a sample of 17 couples (one couple withdrew participation halfway through the study). Ideally this sample should be higher in order to increase statistical power, especially when using a between-subjects design to compare an experimental group to a control group. Also, a control group (either wearing passive bands or using a non-tactile communication device with a similar low bandwidth) is needed to confirm that the increase in social connectedness is a result of the use of the MST technology and not a side effect of the study *per se*. Moreover, the specific study sample used in this study (e.g., couples not living together, both with specific mobile devices) makes it harder to generalize the results of this study to a general population.

Lastly, another limitation of this study is related to the Longing for Interpersonal Touch Picture Questionnaire (LITPQ: Beßler et al., 2019). Variability was high in the longing for touch data among participant couples. As stated earlier, this may be explained by the way the questionnaire is scored (between 0 and infinity). Another reason may be that the LITPQ is a fairly new instrument (developed in 2020) and (further) validation of the questionnaire is needed in order to obtain consistent data with less variability.

### Future Research

The majority of research in the field of MST technology has been conducted in lab settings (van Erp and Toet, 2015; Eid and Al Osman, 2016; Huisman, 2017). The study reported here is an exception in that it is a longitudinal field study that aimed to provide insights into the actual use of MST and potential effects of extended use of this technology on social connectedness and longing for touch. Despite the limitations of the present study, it is to the best of our knowledge the only one that investigated the use of MST technology in a natural setting over a longer period of time. Participants were free to use the technology as they saw fit. This approaches a more naturalistic setting for the application of MST technology and future studies of this kind may help to shed more light on the use of MST technology. More specifically, future studies may focus on comparing different groups (e.g., homogenic and heterogenic couples, using MST technology or not, etc.) and be longitudinal (i.e., longer than 2 weeks) in nature to control for potential novelty effects when first using new MST devices. Since none of the participants in the current study interpreted the haptic bracelets' signal as interpersonal touch, it may also be interesting to observe whether the effects on connectedness and longing for touch are the same when mediated touch is indeed experienced as interpersonal touch.

Furthermore, replication of the results reported here is needed, given the limitations and scope of the present study. Mediated touch should ideally counteract the negative consequences of touch deprivation (and ultimately convey the advantageous effects of real, unmediated social touch). As van Erp and Toet (2015) suggested: mediated social touch must preferably be understood without diminishment of effectiveness and user gratification. The findings of this study suggest that geographically separated romantic couples feel more socially connected through the use of haptic bracelets in a naturalistic setting. However, it may be interesting to see whether a similar effect can be replicated for non-romantic dyads (e.g., friends, acquaintances, strangers). When these effects can be reproduced and supported by the use of a control group, MST technology can potentially be practically implemented in settings where touch is scarce or particularly beneficial (e.g., nursing homes or therapeutic settings).

In addition, mediated social touch is highly contextual (Eid and Al Osman, 2016; Huisman, 2017). The integration of MST technology in a multimodal way (i.e., combined with other sensory input), as well as the addition of options such as warmth and other forms of touch (e.g., stroking), and the application to different body locations may be promising directions for further MST research. When measuring social connectedness and its dimensions, it may be interesting to see how a multimodal approach influences these measures.

Finally, related to the multimodal context that is relevant to MST technology, the embedding of such technology in existing socio-technical landscapes also deserves further scrutiny. The study of MST in naturalistic settings should not only focus on the technology as it is relevant to the communication between, say, couples, but also needs to consider the fact that such technology is used in a broader spectrum of other technologies and media that are already used. As an example, the TA reported here highlighted how the bracelets were used as a low-effort way to say “*I'm thinking of you*,” which complimented the use of other technologies, such as texting. Moreover, MST technologies may also be used during ongoing social interactions in existing social structures, such as meetings in an office. The way MST technologies are situated in such interactions and structures needs to be further investigated. For example, the TA indicated how others noticing the bracelets (e.g., noticing the sound production by the bracelets during a face-to-face meeting) impacted participants' use of the bracelets. Such factors need to be considered in studies of MST technologies in naturalistic settings.

## Conclusion

In this longitudinal explorative field study, the effects of daily use of MST were investigated during a two-week period on social connectedness and longing for touch among geographically separated romantic couples. The results show that overall social connectedness levels (and specifically the dimensions of *relationship salience, feelings of closeness*, and *contact quality*) significantly increased after using MST technology for 2 weeks. Couples' *longing for touch* scores were not significantly different before and after using the bracelets. Furthermore, two main themes were generated from the post-study interview data by way of (reflexive) TA: (a) The haptic bracelet fosters a positive one-to-one connection with partner; (b) Working around frustrations as part of the study. These themes shed further light on the quantitative results. While the increase in social connectedness observed in this study is in line with prior findings in MST research, some caution has to be taken with the interpretation of the results due to technical issues impacting the use of the bracelets. Future research could aim to replicate the findings reported here and also investigate the longitudinal effects of MST technology in different realistic contexts, for different (non-romantic) relationships, and possibly include other sensory modalities like sound, vision or even smell, in line with a multisensory approach. Whether or not this leads to remote interactions that are “*better than a like on Facebook*” remains to be seen.

## Data Availability Statement

The original contributions presented in the study are included in the article/Supplementary Materials, further inquiries can be directed to the corresponding author.

## Ethics Statement

The studies involving human participants were reviewed and approved by TNO Internal Review Board. The patients/participants provided their written informed consent to participate in this study.

## Author Contributions

MH conceived the original idea, performed the experiments, analyzed the data, and wrote the initial draft paper. MH, GH, and AT conducted the interviews and performed the thematic analysis. JE critically reviewed the draft manuscript. All authors were actively involved in the revisions of the original drafts and in writing the final version. All authors contributed to the article and approved the submitted version.

## Funding

This project was partially funded by TNO's Early Research Project Social Extended Reality.

## Conflict of Interest

Between January 2017 and April 2020 GH worked on the development of Hey Bracelet. The remaining authors declare that the research was conducted in the absence of any commercial or financial relationships that could be construed as a potential conflict of interest.

## Publisher's Note

All claims expressed in this article are solely those of the authors and do not necessarily represent those of their affiliated organizations, or those of the publisher, the editors and the reviewers. Any product that may be evaluated in this article, or claim that may be made by its manufacturer, is not guaranteed or endorsed by the publisher.
