# Supplementary material for: Connected Through Mediated Social Touch: “Better Than a Like on Facebook.” A Longitudinal Explorative Field Study Among Geographically Separated Romantic Couples
Source: Front Psychol. 2022 Feb 17;13:817787. doi: 10.3389/fpsyg.2022.817787 (PMC8891523; doi:10.3389/fpsyg.2022.817787)
Supplement: Supplementary file 1 [file Data_Sheet_1.DOCX]

Supplementary Materials

SM - A List of mobile devices tested with the Hey bracelets p. 2

SM - B Questionnaire: Social Connectedness p. 3

SM - C Questionnaire: Longing for Touch (LITPQ) partner subscales p. 4

SM - D Questionnaire: Touch Avoidance (TAQ) partner subscale p. 5

SM - E Questionnaire: Affinity for Technology (ATI) Scale p. 6

SM - F Questionnaire: Extraversion subscale (IPIP) p. 7

SM - G Explorative Questions shown in ‘After Questions’ p. 8

SM - H List of tips for apps and using the bracelets p. 9

SM - I Feedback testing and pilot Hey bracelets p. 10

SM - J Recruitment criteria for participants p. 11

SM - K Recruitment Flyer p. 12

SM - L Information Document p. 13

SM - M Informed Consent p. 16

SM - N Screenshot of initial instructions in the HowAmI app p. 17

SM - O Feedback Hey bracelets by participants p. 18

SM - P Correlations Social Connectedness and Individual Characteristics p. 19

SM - Q Schematic of Inside Hey bracelet p. 21

SM - R Original Dutch Quotes for Thematic Analysis p. 22

**Supplementary Materials – A**

**List of mobile devices tested with the Hey bracelets prior to this study**

iOS devices:

- - - iPhone 7 - iOS 12.4
    - iPhone 6S   - iOS 13.1
    - iPhone 7 - iOS 13.5.1
    - iPhone XS - iOS 13.3
    - iPhone XR – iOS 14.3
    - iPhone 11 Pro Max - iOS 13.5.1

Android devices:

- - - Samsung galaxy S9 - Android 10
    - Samsung galaxy S8 - Android 8
    - Samsung galaxy S8 - Android 9
    - Samsung galaxy S9 - Android 7
    - Samsung galaxy S6 - Android 7
    - Samsung galaxy S10 - Android 10
    - Samsung galaxy S10 - Android 10e
    - Samsung Galaxy S20+ - Android 11.0

**Supplementary Materials - B**

**Questionnaire: Social Connectedness**

| Sensitive and time discriminant measurement of subtle changes in the subjective experience of belonging and relatedness resulting from the use and introduction of different communication systems |
| --- |
| *Relationship salience* 1. Aside from our contact, I often feel "together" with X somehow. 2. I often think of X. 3. Even when we are not in each other’s' company, I often feel "together" with X somehow. 4. I am often aware of my relationship with X.  *Dissatisfaction with contact quality* 1. I derive little satisfaction from my contact with X (R). 2. I feel that X does not understand me well (R).  3. My contact with X feels superficial (R).  *Shared understandings* 1. I feel that X shares my interests and ideas.  2. I feel I have a lot in common with X. 3. I feel on the same wavelength with X.  *Knowing each other’s' experiences* 1. I often know what X feels. 2. I often know what X thinks. 3. I feel that X often knows what I think.  4. I sense that X often knows what I feel.  *Feelings of closeness* 1. In comparison with all your other relationships (with both men and women), how close is your relationship with X? 2. In comparison with what you know of the relationships of other people (with both men and women), how close is your relationship with X? 3. I feel I can talk about anything with X. 4. I feel that X and I can communicate well with each other.  *(R) = Reversed* |

**Supplementary Materials - C**

1. **
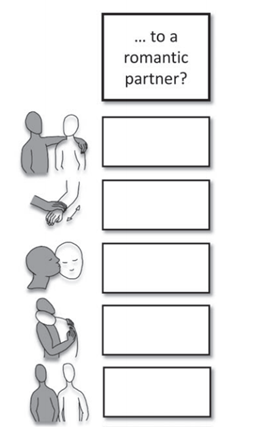
**
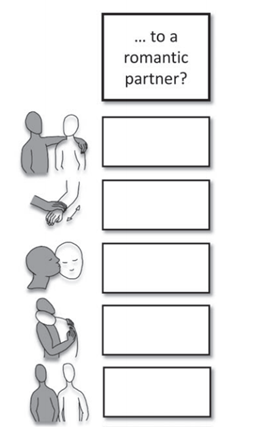
 **B)**

**Supplementary Figure 1.** Longing for Touch Picture Questionnaire (LITPQ) partner subscales, as shown to participants of the study in the HowAmI app. The Questions for this scale were A) *How often did you experience this type of touch in the last two weeks?*, and B) *How often would you have wanted to experience this type of touch in the last two weeks?.*

**Supplementary Materials - D**

**Questionnaire: Touch Avoidance (TAQ) partner subscale**

1. I wish my partner would hold me for hours. (R)
2. Often, I have to tell my partner to cease touching me.
3. For most of the time, I don’t like it when my partner touches me.
4. Sometimes I make efforts to avoid touching my partner.
5. I love to hug and caress my partner for hours. (R)
6. I’m always happy when my partner touches me. (R)
7. Sometimes I find my partner’s touch irritating.
8. My partner often complains that I don’t touch him/her enough.
9. I often find my partner’s touch unbearable.
10. I don’t want my partner to touch me in public.

*(R) = Reversed coded*

**Supplementary Materials - E**

**Questionnaire: Affinity for Technology (ATI) Scale**

Affinity for Technology Interaction (ATI) Scale

Franke, Attig, & Wessel (2018)

| In the following questionnaire, we will ask you about your interaction with technical systems. The term “technical systems” refers to apps and other software applications, as well as entire digital devices (e.g., mobile phone, computer, TV, car navigation). | | | | | | | |
| --- | --- | --- | --- | --- | --- | --- | --- |
| Please indicate the degree to which you agree/disagree with the following statements. | | completely disagree | largely disagree | slightly disagree | slightly agree | largely agree | completely agree |
|  | I like to occupy myself in greater detail with technical systems. |  |  |  |  |  |  |
|  | I like testing the functions of new technical systems. |  |  |  |  |  |  |
|  | I predominantly deal with technical systems because I have to. (R) |  |  |  |  |  |  |
|  | When I have a new technical system in front of me, I try it out intensively. |  |  |  |  |  |  |
|  | I enjoy spending time becoming acquainted with a new technical system. |  |  |  |  |  |  |
|  | It is enough for me that a technical system works; I don’t care how or why. (R) |  |  |  |  |  |  |
|  | I try to understand how a technical system exactly works. |  |  |  |  |  |  |
|  | It is enough for me to know the basic functions of a technical system. (R) |  |  |  |  |  |  |
|  | I try to make full use of the capabilities of a technical system. |  |  |  |  |  |  |

From: [www.ati-scale.org](http://www.ati-scale.org)  *(R) = Reversed*

**Supplementary Materials - F**

**Questionnaire: Extraversion subscale (IPIP)**

| 1. Am the life of the party. |
| --- |
| 1. Don't talk a lot. (R) |
| 1. Feel comfortable around people. |
| 1. Keep in the background. (R) |
| 1. Start conversations. |
| 1. Have little to say. (R) |
| 1. Talk to a lot of different people at parties. |
| 1. Don't like to draw attention to myself. (R) |
| 1. Don't mind being the center of attention. |
| 1. Am quiet around strangers. (R) |

*(R) = Reversed coded*

From: <https://ipip.ori.org/New_IPIP-50-item-scale.htm>

**Supplementary Materials - G**

**
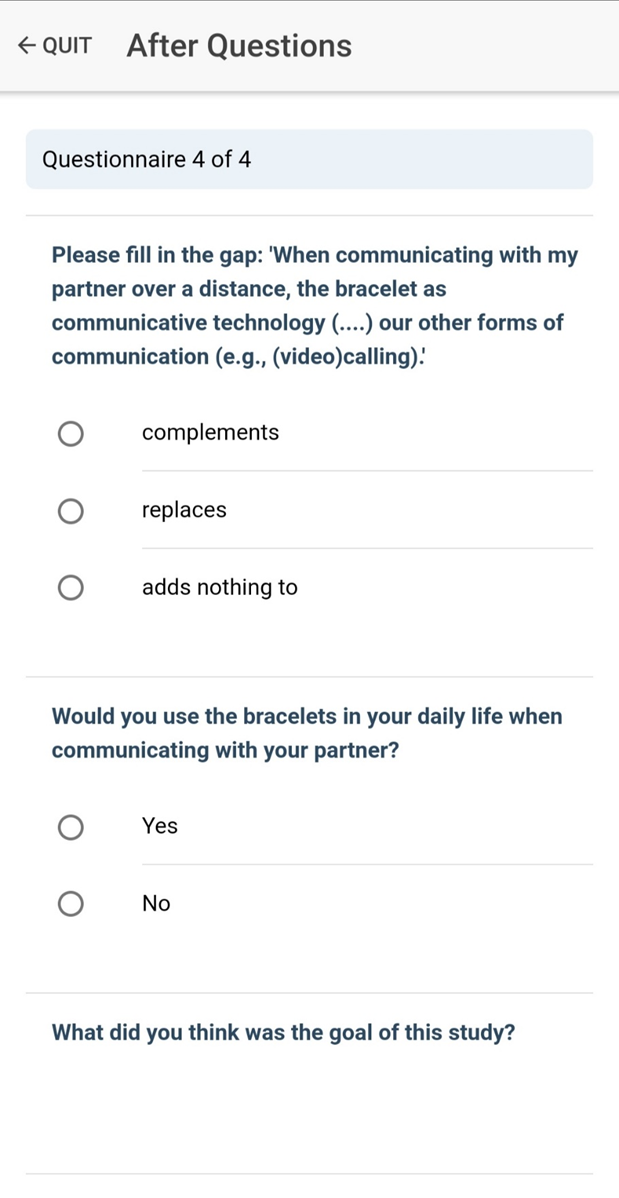
**

**Supplementary Figure 2.** Explorative Questions as shown to participants in ‘After Questions’ in the HowAmI app.

**Supplementary Materials - H**

**List of tips for apps and using the bracelets**

This document was sent to participants along with the Hey bracelets at the start of the study.

Before using the bracelets, please download the apps below according to the following steps:

**HowAmI app:** This app is needed to fill in the questionnaires during this study.

- Download the HowAmI app, developed by TNO, on your mobile device.
- Log in with the following four-digit study code: ____.
- Log in with your specific username and password provided to you.
- Fill in the ‘before questions’ before using the bracelets, the ‘during questions’ every day (14 days total) during the experiment, and the ‘after questions’ after the 14 days of using the bracelets.

**Hey app:** This app is needed to use the bracelets during this study.

- Download the Hey app on your mobile device.
- Log in with the same email address that you filled in on the TNO website and the specific password given to you along with the bracelet. At the end of the study, the experiment leader will use these details to obtain user data of the bracelets (frequency of touches).
- Update the Hey app to v.1.7.3 and the bracelet to firmware v1.01 before usage.

**DontKillMyApp app:** Android users specifically need to download this app on their smartphone.

- This app gives instructions tailored to your specific device on how to avoid unwanted disconnection of the bracelets and closing of the apps involved in this study due to background battery management protocols.
- Please follow the instructions as given by this app before using the bracelets. The most important part is that the *Hey* and *HowAmI* apps get full permission in your settings and will not be ‘optimalised’ for your battery.
- iPhone users do not need to download DontKillMyApp, since IOS devices have less aggressive battery management protocols compared to Android devices.

**Additional information and tips for using the bracelets:**

- When using the bracelet for the first time, try to adjust it in a way it feels comfortable. If it is too tight or too loose, touches may feel uncomfortable.
- To avoid (unwanted) disconnection, please try to keep the bracelet as close to your mobile phone as possible (maximum of 10 meters between phone and bracelet).
- The bracelet is weatherproof, not waterproof. For example, do not shower while wearing the bracelet.
- The bracelet is made of plastic and silicon.

**Supplementary Materials - I**

**Feedback testing and pilot Hey bracelets prior to the current study**

**First testing period:**

- Person 1: Samsung Galaxy S20+ with Android v11.0. – Only worked properly after changing phone settings according to advice given by the DontKillMyApp app.
- Person 2: iPhone XR with iOS v14.3. -Worked excellent.
- Bracelets both had firmware v1.01 and Hey app v1.7.3.

**Pilot:**

- Person 1: iPhone 11 with iOS v14.4. -Worked excellent.
- Person 2: Huawei p10 Lite met Android v8.0.0. – Did not work properly, even after changing phone settings according to the DontKillMyApp app and multiple benchmarks.
- Bracelets both had firmware v1.01 and Hey app v1.7.3.
- This couple sent a lot of unwanted/accidental touches to one another. For example, when putting on the bracelets, or when the bracelet touched a leg when sitting on the couch. One person even sent 6 unwanted touches over a period of 2 minutes.

**Supplementary Materials - J**

**Recruitment criteria for participants**

**Inclusion criteria:**

- Romantic couples.
- Between 18 to 65 years of age.
- English proficiency (since the questionnaires will be in English).
- Owning an iPhone (preferred), Sony Xperia, or Samsung Galaxy S series.
- Agrees to share user data of the bracelets (e.g. time and frequency of bidirectional touches) within the Hey app at the end of the study, in order to take various exploratory factors into consideration in the analyses. This data will be pseudo-anonymized before analyses and storage.

**Exclusion criteria:**

- Living together with partner (to stimulate engagement with the bracelets).
- People that are strongly aversive to technology.
- People that are strongly aversive to touch.
- People with physically demanding professions (e.g. construction workers), since they probably cannot wear the bracelets as much as intended/needed.

**
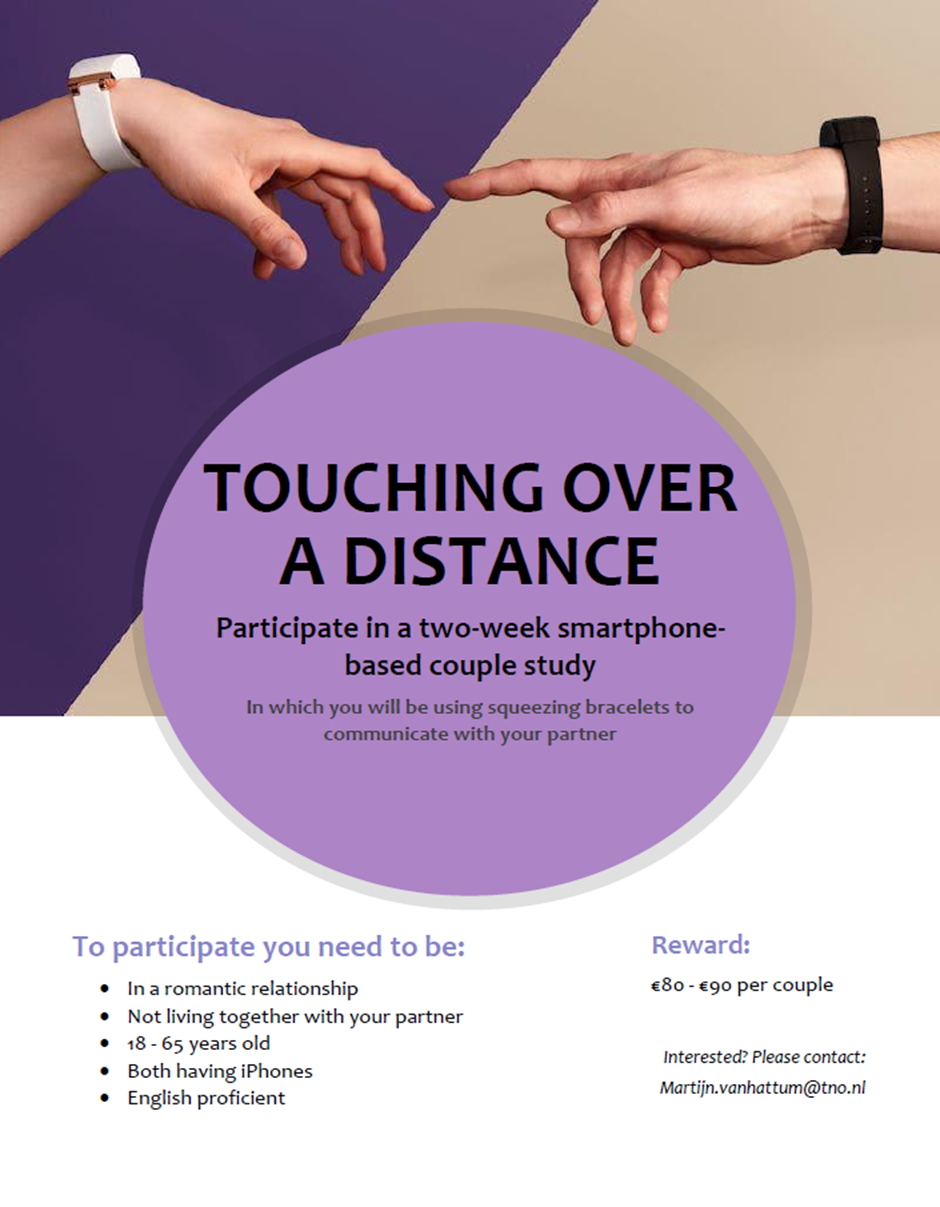
Supplementary Materials - K**

**Supplementary Figure 3.** Recruitment Flyer for the study

**Supplementary Materials - L**

**Information document**

1. **What is the goal of the research?**

Nowadays, a big part of interpersonal communication happens over a distance. This communication mostly happens through vision and sound (e.g., (video-)calling), while less remote communication happens through other senses. In this study, remote communication among geographically separated couples is investigated via squeezing bracelets. With the information obtained in this study, the functionality of the bracelets and comparable technologies can be improved. Your participation in this study is of great importance to gain insight into remote communication among couples via squeezing bracelets in a naturalistic setting.

1. **About TNO**

De letters TNO stand for Dutch Organisation for Toegepast Natuurwetenschappelijk Onderzoek (Applied Nature-scientific Resereach). TNO develops knowledge targeting practical application and focuses on the following areas of interest: Construction, Infra & Maritime; Circular Economy & Environment; Defense, Safety & Security, Energy; Industry; Healthy Living; Strategic Analyses & Policy; Mobility & Logistics; Information & Communication Technology. The current research falls under the domain Defense, Safety & Security, which aims to create a safer society by creating innovations for people who are daily committed to security in defense, police, emergency services and commercial businesses.

1. **Participation in research study**

You are explicitly asked to participate in this study. You are contacted because you fit in to the target population of the current study, which is why we hope you will participate. All experimental data in this study will be obtained by (online) questionnaires which can be filled in in an app on your mobile device. Participation will take up two weeks and the study will take place in your personal setting (so it is not needed to travel to a testing location). A total of twenty participant couples will be recruited in this study.

1. **Who can participate in this study?**

There are several requirements in order to participate in this study. First, only couples between 18 – 65 years, who do not live together can participate. Second, people with specific mobile devices can participate in order to minimalize technical issues with the bracelets. iPhones are the preferred device, but some Android devices (such as Samsung Galaxy S series) may also participate. Furthermore, participants need to be English proficient since the questionnaires will be in English. Participants also need to be willing to share data from the Hey app (time and frequency of touches) with the experiment leaders at the end of the experiment. Couples that live together, people who are (strongly) aversive to touch and/or technology, and people with professions that do not allow to wear the bracelets (properly) are all excluded from participation.

1. **How will the study be conducted?**

For this study, the effects of remote communication through bracelets among couples will be investigated over a period of two weeks. You and your partner will both receive bracelets which can be used to send each other ‘touches’ via squeezes. This study will be conducted in your own time and place.

1. **Wat is expected of you?**

You are expected to communicate with your partner using the squeezing bracelets for two weeks, in the way you both want. This study will thus take place in a time and place of your choice. You are only asked to both use the bracelets. To participate, you will need to download (at least) two apps on your mobile device. The *Hey* app need to be downloaded to use the bracelets and connect your bracelets to your partners’ bracelet.

To fill in the questionnaires for this study, participants will need to download the *HowAmI* app. This app is in-house developed by TNO and will ensure safe data collection and storage. The questionnaires in this study need to be filled in on various moments. Before and after using the bracelets with your partner for two weeks, two questionnaires will be presented in the *HowAmI* app (approximately 9 and 6 minutes respectively). During the study, you will be asked to answer a small number of questions at the end of each day (1 minute). Furthermore, you are asked to participate together with your partner in an interview at the end of the study. With information gained from these interviews, the technology used in this study can be improved.

Apart from the *Hey* and *HowAmI* apps, participants with Android devices are required to download a third app (*DontKillMyApp*) before using the bracelets. This app will provide instructions on how to change the battery settings of your Android device in order to optimalize the user experience with the *Hey* and *HowAmI* apps, as well as the bracelets. Participants with iPhones are not required to download the *DontKillMyApp* app.

For this study, you are asked to give up your address in order to send the bracelets you. Your email/phone number are asked to keep in contact with the experiment leader during the study. You are asked to give up your bank details in order to receive your reward at the end of the study. Your permission is asked to share the user data (frequency and location of send/received touches) of the bracelets with the experiment leader at the end of the study. For this study, data will be pseudo-anonymized and all information shared by participants will be used confidentially, so no information can be retraced to participants. The obtained data will be stored on secure TNO servers and personal data will be deleted at the end of the study.

1. **What are potential (dis)advantages for participating in this study?**

There are no expected disadvantages for participating in this study.

1. **What happens when you do not want to participate (any more) in this study?**

Your participation in completely voluntary and can be withdrawed without reason at any time and without any consequences.

1. **What happens with your details?**

TNO greatly values your privacy and considers the applicable privacy rules. Numerous measures have been taken to protect your data. The data is processed within well-secured computer systems to which unauthorized persons have no access. To guarantee your privacy, your name and contact details are kept separate from your research data as much as possible. TNO guarantees that your data will only be used for research purposes. Your data is stored on a separate TNO server and is only accessible to authorized members of the research team. Third parties do not have access to the collected data. Inspection by authorized inspectors may be required to verify the reliability and quality of the investigation. In publications about the research (the answers of) individual participants are in no way recognizable. After the research has ended, your research data can be kept for 10 years. Based on privacy legislation, you have rights such as the right to inspect, delete your data or correct your personal data. More information about which rights apply and how you can apply them can be found in the privacy statement (https://www.tno.nl/nl/over-tno/contact/corporate-legal/privacystatement/) of TNO. You can also send an e-mail about this to privacy@tno.nl.

1. **Is there be compensation when you decide to participate in this study?**

For participating in this study, every participant will receive a compensation of at least €40. A bonus of €5 (total of €45) can be earned when filling in at least 75% of all questionnaires in this study. This compensation will be transferred directly after the study to the bank account specified by you. TNO is obliged to report the given compensation to the tax authorities.

1. **Ethical aspects**

TNO treats you with care. U participate voluntarily and will be told exactly what to do when participating. If you agree and you are eligible to participate, you will start the study. You can also stop participation during the study if you do not like it (anymore). You do not need to state a reason. This study is conducted in accordance with all applicable national and international legislation and guidelines aimed at monitoring your health and safety. The research was tested in advance by the internal review committee for human-related research of TNO.

1. **Insurance**

TNO has insured everyone participating in this study. The insurance covers damage resulting from participation in the study. Damage must be reported to TNO as soon as possible.

1. **Would you like to know more?**

For any questions before, during, or after this study, you can contact Martijn van Hattum.

M: +316__________

E: martijn.vanhattum@tno.nl

**Supplementary Materials - M**

**Informed Consent**

Undersigned,

Name: ____________________________________________________

Date of birth: _____________________________________________________

declares to participate in the study on a voluntary basis, entitled:

*Connecting through mediated social touch* at TNO.

- I confrm that I have read the information on the study mentioned above.
- I understand the information.
- The intentions of the study and the followed approach have been explained to my satisfaction.
- I have had the opportunity to ask additional questions and these questions have been answered satisfactorily.
- I have had enough time to think about participation .
- I know that my participation to this study is completely voluntary and I can withdraw my consent at any time without having to give a reason.
- I give permission to process my personal data for the purposes as described in the information.
- I give permission to reuse my research data for future research in the described research area, on the condition that it is coded in such a way that it cannot be traced back to me as a person.
- I give permission for storage of the data and that authorized members of the investigation team and authorized inspectors have access to it.
- I consent to be interviewed about my experience with the technology (Hey bracelets) at the end of the study.
- I declare that I have no known impediments to participate in this study.

Place, Date: ____________________________________

Signature participant: ____________________________________

**Supplementary Materials - N**

**
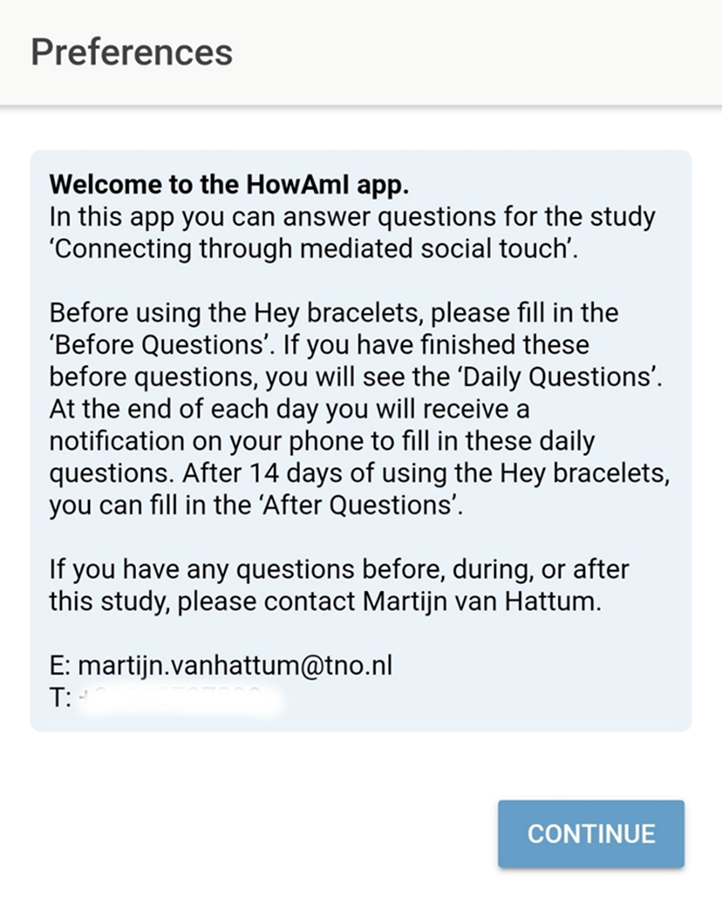
**

**Supplementary Figure 4.** Screenshot of the Initial Instructions in the HowAmI app, as shown to participants of the study.

**Supplementary Materials - O**

**Feedback Hey bracelets by participants in the current study**

- Battery life for the bracelets was very inconsistent among participant couples (some needed to charge at least every day, some every 4 days)
- Couple #3 had multiple connection issues with both bracelets.
- Couple #5 had a technical issue where the bracelet made a lot of noise and did not function properly (as shown in a video recording of the bracelet) **🡪 sent a new bracelet**
- Couple #6 had problems connecting with 1 bracelet, very inconsistent when trying to pair. After consultation, connection was better.
- Couple #7 accidentally swam while wearing the bracelet **🡪 sent a new bracelet**
- Couple #8 had connection issues with the bracelets **🡪 sent a new bracelet. Even after this, 1 of the phones (Samsung Galaxy S20) didn’t function properly with the bracelet.**
- Couple #10 lost one of the bracelets. **🡪 called the participant, decided to send a new bracelet after consultation**
- Couple #12 stopped the experiment mid-way due to connection issues between the bracelet and their phones (both Sony Xperia). This was potentially expected at the start of the study, since the bracelets were not tested before on Sony Xperia’s.
- Couple #14 had one bracelet that initially worked, but after some days stopped working and did not react to anything anymore. **🡪 sent a new bracelet**
- Couple #16 emailed me that one bracelet broke down when it fell and someone stepped on it **🡪 sent a new bracelet**.
- Couple #17 emailed me that installing the bracelet was hard and that a lot of touches that were send were unintentional. After some days it seemed that it was one particular bracelet that gave the issues, while the other one worked well **🡪 sent a new bracelet**
- Couple #18 had consistent connection issues with 1 bracelet **🡪 sent a new one**

Nearly all connection issues in the experiment derived from failures in the Bluetooth connection between the Hey bracelet and the mobile phones. Even after changing (battery)settings following the *DontKillMyApp* list, these problems persisted. Even participants with iPhones experienced problems in the connection between phone and bracelet, even though this seemed to work well in the pilot study.

- Total amount of new bracelets send to participants: 8 (out of 38 total) = 21,05% of total
  - Of which 5 bracelets (13,16% of all bracelets) that had to be replaced due to technical issues.


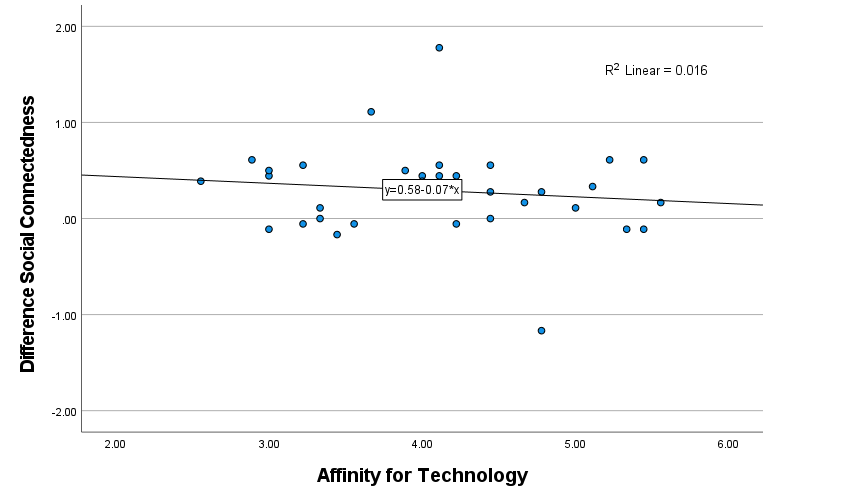
**Supplementary Materials – P**

**Supplementary Figure 5.** Pearson’s Correlation between the Difference scores of Social Connectedness (post-score – pre-score) and Affinity for Technology Scores of Individual Participants. The Correlation was not Significant, *p* = .915


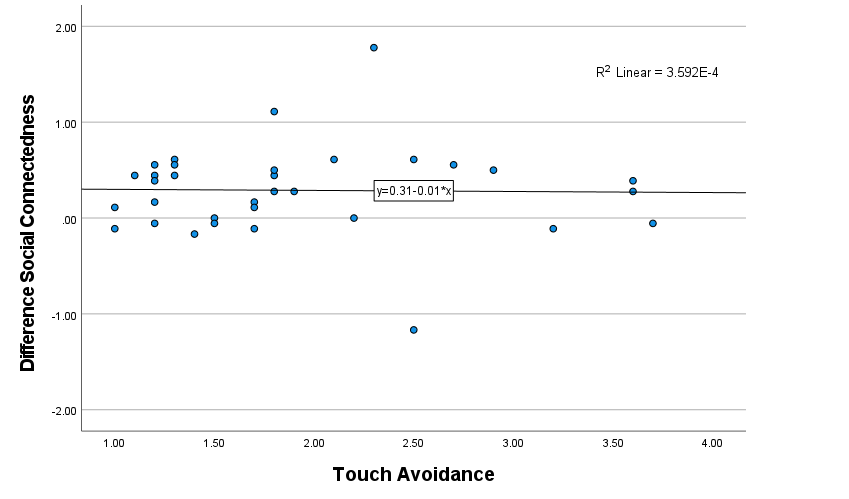


**Supplementary Figure 6.** Pearson’s Correlation between the Difference scores of Social Connectedness (post-score – pre-score) and Touch Avoidance Scores of Individual Participants. The Correlation was not Significant, *p* = .47

**
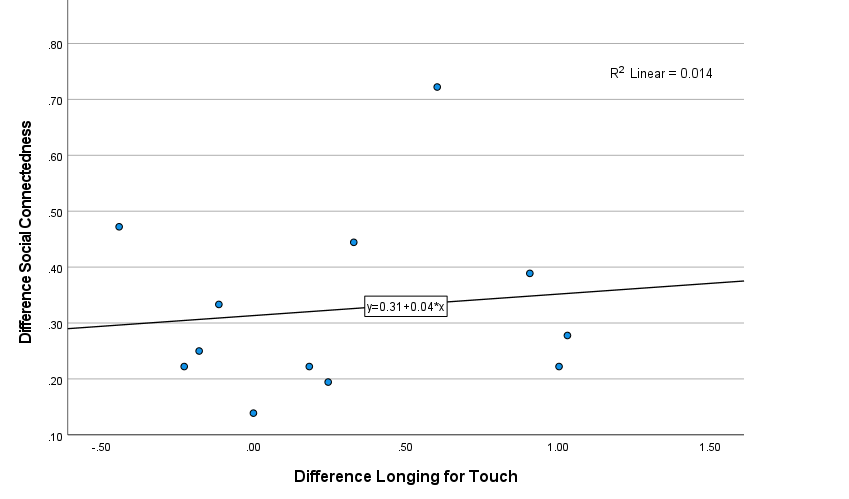
**

**Supplementary Figure 7.** Pearson’s Correlation between Couples’ Difference scores (post-score – pre-score) of Social Connectedness and Longing for Touch. The Correlation was not Significant, *p* = .71.

**Supplementary Materials – Q**


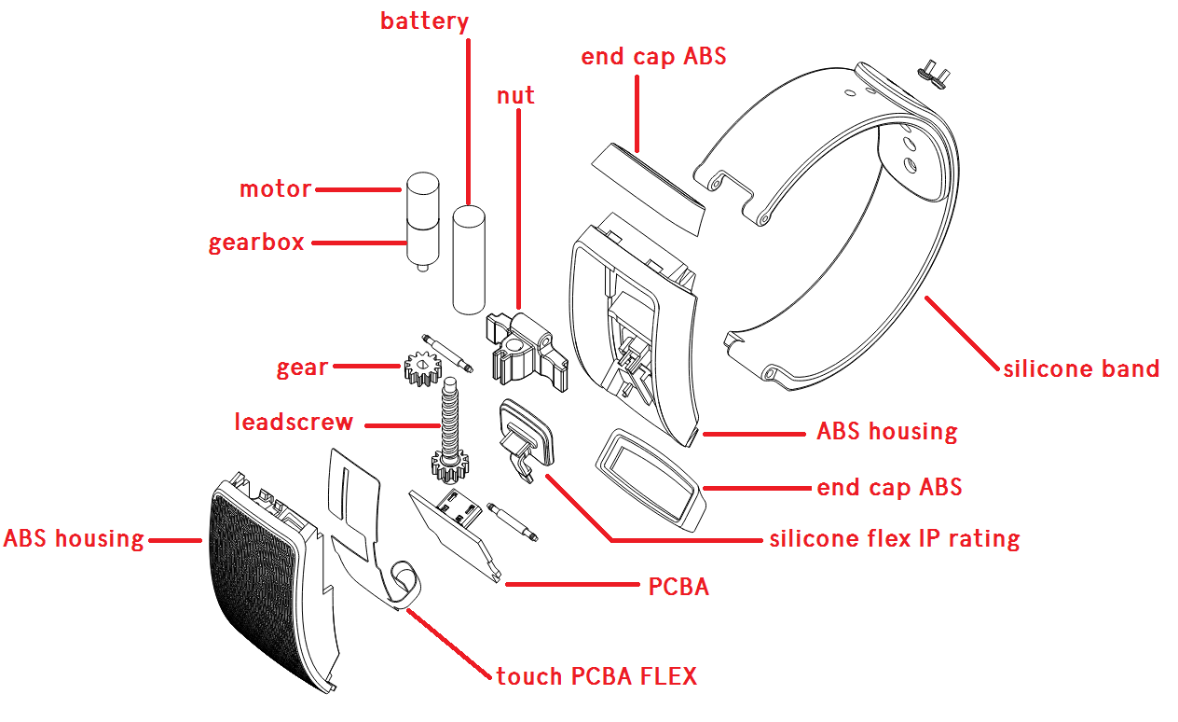


**Supplementary Figure 7.** Schematic of the inside of the Hey bracelet

**Supplementary Materials – R**

**Original Dutch interview quotes for Thematic Analysis**

**Theme 1: The haptic bracelet fosters a positive one-to-one connection with partner**

“Ik was heel erg bewust dat ik dat bandje om had en wat er gebeurt op het moment dat die afgaat, dus zodoende kon ik het niet vergelijken met een echte aanraking. Maar de gedachte erachter, dat maakt het wel fijn; Dat ik weet dat zij het heeft gedaan, zij stuur het, dus het is meer het contact wat erachter zit dat het fijn maakt voor mij.” {Couple 18, PP2}

“Ja, ik moet zeggen omdat je weet dat die aanraking van je partner komt, daardoor verzin je er zelf bij dat het een aanraking is.” {Couple 1, PP1}

“Ook dat je precies weet dat er is niemand anders is die dit doet. Want je telefoon kan ook trillen van iemand anders. En bij dit [signal Hey bracelet] weet je gewoon zeker dat het NAAM was, zegmaar. En dan is dat wel zo'n fijn gevoel.” {Couple 3_PP2}

“Ja en zeker ook omdat we normaal gesproken, zeker in de periode dat we de armbandjes gebruikten, was hij gewoon heel veel in CITY1 en ik heel veel CITY2, dus je mist elkaar dan toch wel gewoon echt en je mist het kunnen knuffelen en het af en toe even een kus kunnen geven. En dat [using Hey bracelets] was een manier en voelde het toch dichterbij. Ja, dus, de eerste keer dat ik hem niet om had na het onderzoek, toen was ik ook helemaal van, nou…” {Couple 11_ PP1}

“Ja, voor mij betekende het [signal bracelet] niet eens ‘Ik ben er’, maar vooral ‘Ik denk aan jou’ . Ze weet dat ik aan haar denk; Dat ik gewoon even wel met haar bezig ben in plaats van mezelf. Ik denk dat dat heel fijn is. Als iemand gewoon de tijd neemt tussen alles wat hij dan doet, hoe druk je dag is en noem maar op, toch even “Ik denk aan je” en dat dan sturen. Meer niet. Ik denk dat dat heel, heel prettig is.” {Couple 18_ PP2}

“Ik had aan het begin eerst van: NAAM heeft een touch gestuurd, dus ik stuur hem terug, zeg maar. Maar het is natuurlijk niet een soort van morsecode naar elkaar of zichtbare communicatie, dus op een gegeven moment stop je daar wel mee en is het wat meer op momenten dat je aan de ander denkt. Ik denk dat je het dan stuurt.” {Couple 17_PP2}

“Nou, ik koppel dit eigenlijk niet eens zozeer aan een aanraking ofzoiets. Het was voor mij meer, bijvoorbeeld als ik s morgens wakker werd en NAAM stuurde mij een touch dat ik dacht ‘hé, hij denkt aan me, wat leuk’, en niet perse zozeer een aanraking.” {Couple 10, PP1}

“Ik vond het vooral fijn als ik dan zelf bijvoorbeeld een beetje in de put zat, dat je dat [touch signal] dan even kan sturen en dat je daarmee inderdaad ook om een soort van aandacht kan vragen van ‘Ik heb nu gewoon even wat nodig’. En, dat kon dan op die manier makkelijker.” {Couple 11_ PP1}

“Dat ik eigenlijk zin had om een touch te krijgen, maar die kreeg ik niet. Dus dan stuurde ik er een en dan krijg je hem alsnog. Een soort aandacht.” {Couple 6, PP2}

“Ik geef hem wel 9 van de 10 keer terug.” {Couple 3_PP1}

“Het voelde heel raar om niet dat [sending a touch signal] terug te doen.” {Couple 9_PP1}

“Soms wilde ik laten weten dat ik aan hem dacht, en soms wilde ik zelf ook even aandacht; Dan wilde ik dat hij aan mij dacht, dus dan stuurde ik een touch, en dan hoopte ik dat hij iets terugstuurde. Ik merkte dat ik dat soms wel moeilijk vond als dat dan niet gebeurde, want ik wilde dat dan wel heel graag terugkrijgen. Maar ja, misschien is iemand net bezig of zo.” {Couple 16_ PP1}

“Op het moment dat je aan iemand denkt, hoef je niet op je telefoon te kijken ofzo. Dus dat gebruiksgemak is er dan wel.” {Couple 12, PP2}

“(…) Ik vond het soms wel heel handig, bijvoorbeeld als je even geen tijd had om een berichtje te sturen omdat je daar toch meer over na moet denken en je een berichtje moet typen, dan kan je wel even je hand op dat bandje leggen en op die manier iemand laten weten dat je aan diegene dacht. Dus dat vond ik wel een hele leuke meerwaarde.” {Couple 16, PP1}

“Als ik geen tijd had om een appje te sturen ofzo, of je was gewoon aan het werk.. Ja, Ik werk met mensen, met gasten enzo, dus dan kan ik niet echt mijn telefoon oppakken. Dus dan was een touch sturen net even wat makkelijker dan wanneer ik een appje zou sturen.” {Couple 5_PP1}

“Nee, ik pak niet mijn telefoon om alleen een appje te sturen. Dan is het veel makkelijker om gewoon even je hand er op te leggen bijvoorbeeld. En het kon ook gewoon tijdens het praten of zo. Bijvoorbeeld dat je met iemand praten was en dan sneaky zo je hand op je pols legt.” {Couple 3_PP2}

**Theme 2: Working around frustrations as part of the study**

“Maar doordat we dus van die problemen hadden met de Bluetooth, dan wordt het op een gegeven moment bijna een moeite om het [the bracelet] te gaan gebruiken, maar we deden het natuurlijk ook een beetje voor jullie. We wisten dat we aan een onderzoek meededen, dus toen werd het gebruik [of the bracelets] ook een beetje rommelig.” {Couple 16, PP1}

“Ik vond het [accidental touches] ook niet zo erg, omdat ik er op gegeven moment rekening mee hield. Zo van: dan heb je misschien een keer 1 touch en de andere keren zijn allemaal ongelukken. Maar weet je, dan weegt voor mij die ene [intended] touch nog steeds zwaarder dan die andere 9 die misschien onbedoeld waren, omdat er op een gegeven moment wel bedoeld contact is geweest. Het is niet zo dat als dat ding [bracelet] gewoon op tafel ligt, dat hij dan alleen de touches gaat sturen.” {Couple 18_PP2}

“Ik schrok me gewoon telkens helemaal kapot als dat ding [bracelet] afging. Op de eerste dag heb ik gewoon een kop koffie over het toetsenbord gegooid, omdat ik zo schrok.” {Couple 18, PP1}

“Ik heb me een paar keer echt rot geschrokken van dat ding. Hij heeft een soort stille stand, maar het duurde een paar dagen voor mij om dat te ontdekken. Ik heb hem wel vooral met werk om gehad en echt zeg maar in meetings geschrokken omdat dat ding afgaat. Op een gegeven moment wen je er aan en dacht je van, oh ja, dat is van die app, maar soort van het volume en de onverwachtheid ervan, zeg maar, dat is wel gek natuurlijk.” {Couple 17_PP2}

“Als ik echt ergens helemaal mee bezig ben, dan wil ik altijd volle focus. En als dat ding [bracelet] dan afgaat en ik schrik dan inderdaad, dan ben je weer even van ‘ho’. Ja, het is niet lang ofzo, maar je moet dan toch even terugkomen in waar je mee bezig was.” {Couple 3_PP1}

“Nou, ik had hem [bracelet] niet om als ik ging videobellen, wat nu natuurlijk heel veel gebeurt voor studie, omdat het wel een geluidje maakt. Dus ik dacht van ja, dan zit je in een professionele setting en dan hoor je dat geluid; Nee, dan gebruikte ik hem niet.” {Couple 1_PP1}

“Ik deed hem [bracelet] wel af als ik in een vergadering zat. Dat was meer omdat ik dacht van: ‘als die nu af gaat, dat zou een beetje onhandig zijn’.” {Couple 4_PP2}

“Als ik eerlijk ben, was het wel heel ongemakkelijk met sommige mensen, sommige collega’s.” {Couple 3_PP1_24}

“Het geluid van de bandjes was niet heel discreet. Als je in een ruimte zat, dan wist iedereen ook dat je een aanraking kreeg. Iedereen zat dan wel te staren.” {Couple 2, PP1}
